# Rapid Screening of 352 Pesticide Residues in Chrysanthemum Flower by Gas Chromatography Coupled to Quadrupole-Orbitrap Mass Spectrometry with Sin-QuEChERS Nanocolumn Extraction

**DOI:** 10.1155/2022/7684432

**Published:** 2022-06-15

**Authors:** Yuanyuan Wang, Zhijuan Meng, Chunyan Su, Sufang Fan, Yan Li, Haiye Liu, Xuan Zhang, Pingping Chen, Yunyun Geng, Qiang Li

**Affiliations:** ^1^Department of Pharmacology, College of Basic Medicine, Hebei University of Chinese Medicine, Shijiazhuang, 050200 Hebei, China; ^2^Hebei Food Inspection and Research Institute, Key Laboratory of Food Safety of Hebei Province, Shijiazhuang, 050091 Hebei, China; ^3^School of Pharmacy, Hebei University of Chinese Medicine, Shijiazhuang, 050200 Hebei, China

## Abstract

To analyze pesticide residues, GC coupled with quadrupole-Orbitrap MS (GC-Orbitrap-MS) has become a powerful tool because of its unique characteristics of accurate mass full-spectrum acquisition, high resolution, fast acquisition rates, and overcoming matrix interference. This paper presents an efficiency evaluation of GC-Orbitrap-MS for identification and quantitation in the 352 pesticide residues analysis of chrysanthemum flowers in full-scan mode. A streamlined pretreatment approach using one-step extraction and dilution was used, which provided high-throughput processing and excellent recovery. The samples were extracted using acetonitrile. The extracted solution was purified by a Sin-QuEChERS Nano column to suppress the matrix in chrysanthemum flowers and determined by GC-Orbitrap-MS. The calibration curves for the 352 pesticides obtained by GC-Orbitrap-MS were linear in the range of 0.5–200 *μ*g·kg^−1^, with the correlation coefficients higher than 0.99. The limits of detection (LODs) and the limits of quantification (LOQs) for the 352 pesticide residues were 0.3–3.0 *μ*g·kg^−1^ and 1.0–10.0 *μ*g·kg^−1^, respectively. The average recoveries in chrysanthemum flower at three levels were 95.2%, 88.6%, and 95.7%, respectively, with relative standard deviations (RSDs) of 7.1%, 7.5%, and 7.2%, respectively. Lastly, the validated method and retrospective analysis was applied to a total of 200 chrysanthemum flower samples bought in local pharmacies. The proposed method can simultaneously detect multipesticide residues with a good performance in qualitative and quantitative detection.

## 1. Introduction

Chrysanthemum flower (*Dendranthema grandiflora*) is one of the most common Chinese herbal medicines, and it has been consumed as food for health care and disease prevention since ancient times. It is mainly used for the treatment of respiratory and cardiovascular diseases and shows significant activities, such as antimicrobial, anti-inflammatory, and anti-cancer and neuroprotective and cardiovascular system [1]. Because of its efficacy in alleviating chronic diseases, some consumers often drink chrysanthemum tea as a health food [2]. The chrysanthemum flower, as a good product of the “integration of medicine and food” [3], has a huge consumer group.

However, in order to minimize the loss of crops during planting, pesticides are widely used to control many plant diseases and insects, such as gray mold, rust, aphids, thrips, leaf pickers, leaf folding insects, and spider mites [4]. Therefore, chrysanthemum flowers may be exposed to a variety of pesticides and contain pesticide residues. However, with the widespread use of pesticides, overuse, abuse, and misuse of pesticides also occur from time to time, which will lead to pesticide residues in chrysanthemum flowers, thus constituting a potential threat to human health and adversely affecting international trade.

Because of the potential of pesticide contamination in current agricultural products, many countries and world organizations (e.g., Codex Alimentarius Commission, European Union (EU), United States, Japan, China, Republic of Korea, Canada) have prescribed stringent stipulations for maximum residue limits (MRLs) for pesticides. For instance, there are 162,248 MRL items that cover 465 pesticides in the EU, 39,147 MRL items that cover 351 pesticides in the United States, and 51,600 MRL items that cover 579 pesticides in Japan, and the residue limit level is as low as 10 *μ*g·kg^−1^, and 10,092 MRLs for 564 pesticides in 376 kinds of food was stipulated in China National standards for food safety. Implementing these laws and regulations has strengthened supervision over pesticides and ensured the standard use of pesticides to protect human health. However, laws and regulations of such a multitude of MRLs pose a new challenging issue for the monitoring and controlling of pesticide residues.

At present, the commonly used pesticide residues pretreatment methods include solid-phase extraction [5], solid-phase microextraction [6], gel permeation chromatography [7], and the QuEChERS [8–10]. Among them, the QuEChERS method is widely used, but in most studies, there are many kinds of purification materials with a large amount, low purification efficiency, and large matrix effect, which is not conducive to the rapid and accurate analysis of the experiment [11, 12]. Therefore, the selection of suitable purification materials is conducive to the high-throughput treatment of complex matrices. Sin-QuEChERS Nano column is a new type of rapid sample pretreatment purification column developed and optimized based on the QuEChERS method. Based on the basic principle of reversed dispersion solid-phase extraction, multiwalled carbon nanotubes (MWCNTs), PSA, and C_18_ solid-phase materials are filled into the column tube to achieve one-step purification. MWCNTs have the characteristics of the nanoscale hollow tubular structure and large specific surface area, small dosage, strong adsorption capacity, stability, and durability, which are suitable for the treatment of complex matrices and have better purification and adsorption effect [13–15].

In the past 10 years, most pesticide food control laboratories have shifted from GC-MS to GC-MS/MS as the preferred analytical technology for the treatment of GC amenable compounds. The main reason for this change is that the interference of eluting matrix compounds has a negative impact on single-stage GC-MS analysis. In recent years, the demand for nontargeted detection methods of LC and GC combined with full-scan (FS) MS is increasing, so as to better cover the scope of pesticides and detect them more easily. In GC-MS, FS measurement has been realized for decades, but quadrupole (Q), ion trap, nominal mass time of flight (TOF), and early-generation high-resolution TOF instruments lack sensitivity and/or selectivity. The improvement of high-resolution mass spectrometry (HRMS) in resolution and obtaining appropriate selectivity by improving mass resolution provides new opportunities for residue analysis. Initially, GC and Q-TOF instruments were coupled through the atmospheric pressure chemical ionization (APCI) interface to achieve this goal [16–18], but recently, a special GC and electron ionization (EI) Orbitrap MS system was introduced. The system combines the peak capacity and chromatographic resolution of gas chromatography with the sub-ppm mass accuracy of the Orbitrap system to provide higher resolution (15,000, 30,000, 60,000, and 120,000 at half-maximum (FWHM) at *m*/*z* 200). The collected data are traceable, which is convenient for retrospective analysis and screening of more interested unknown compounds. Compared with the instrument based on APCI, EI is a more general ionization technology. Because multiple ions for quantification and identification can be obtained in one scanning event, the acquisition is also simpler.

In this study, the potential of GC-Orbitrap-MS in nontarget full-scan independent acquisition mode was evaluated for identification and quantitation purposes. A total of 352 pesticides were selected as the target analytical compounds from the 2020 edition of Chinese Pharmacopoeia. An improved QuEChERS method based on Sin-QuEChERS Nano column purification was used [19, 20]. Method validation in chrysanthemum flowers samples was carried out in terms of sensitivity, linearity, ME, and LOQ. Lastly, the validated method and retrospective analysis was applied to a total of 200 chrysanthemum flower samples bought in local pharmacies (Shijiazhuang, China). This method is suitable for the rapid screening and quantitative analysis of multipesticide residues in chrysanthemum flower and provides data and technical support for the safety evaluation of chrysanthemum flowers.

## 2. Materials and Methods

### 2.1. Instruments and Reagents

Analytical standards of the 352 pesticides (10 *μ*g·mL^−1^) were purchased from Alta Scientific Ltd. (Tianjin, China). A total of 352 kinds of pesticide mixed standard stock solution were prepared with acetonitrile at a concentration of 10 *μ*g·mL^−1^ and stored in the refrigerator at −18°C. HPLC-grade acetonitrile was purchased from Merck (Darmstadt, Germany). HPLC-grade water was from Milford Super Pure Water System (Milford, MA). Two types of traditional QuEChERS purification were purchased from Thermo Fisher Scientific (Massachusetts, USA). QuEChERS purification package (simple matrix), includes 50.0 mg PSA and 150.0 mg MgSO4; QuEChERS purification package (complex matrix), includes 50.0 mg PSA, 150.0 mg MgSO4, 50 mg C18, and 50 mg GCB. We used two kinds of salting-out for the QuEChERS method: an unbuffered salt system, including 6 g MgSO_4_ and 1.5 g NaCl, and an acetate buffer salt system, including 6 g MgSO_4_ and 1.5 g sodium acetate, purchased from Thermo Fisher Scientific Inc. (Fair Lawn, NJ). Sin-QuEChERS Nano column, including 2 g Na_2_SO_4_, 0.6 g MgSO_4_, 90 mg PSA, 10 mg C18, and 15 mg MWCNTs, were purchased from China Agricultural University (Beijing, China). All the chrysanthemum flower materials were purchased from local pharmacies (Shijiazhuang, China).

### 2.2. Sample Extraction Methods

The samples were crushed by FW100 High-Speed Universal Crusher (Tianjin Tester instrument Co. Ltd.) and mixed well. Two grams of chrysanthemum flower powder (±0.01 g) were then added into a 50 ml plug centrifuge tube; 10 ml of water was added for redissolution; the tube was vortexed for 1 min; and then the sample was allowed to fully soak and evenly disperse. Then, 10 ml acetonitrile was added, mixed well, and vortexed for 1 min. Next, an acetate buffer salt system containing 6 g anhydrous MgSO_4_ and 1.5 g sodium acetate was added; the tube was vortexed for 1 min, put into an ice water bath for 10 min, and centrifuged for 2 min at 4°C, 9,500 r·min^−1^, and the supernatant was taken for use.

### 2.3. Sample Purification Methods

1. Traditional QuEChERS purification: we tested the purification efficiency of two QuEChERS purification packages: one was a simple matrix, including 50.0 mg PSA and 150.0 mg MgSO_4_, and the other was a complex matrix, including 50.0 mg PSA, 150.0 mg MgSO_4_, 50 mg C18, and 50 mg GCB. We transferred 2 ml of the extracted supernatant into a QuEChERS purification centrifuge tube, mixed this by oscillation for 1 min, centrifuged it at 9,500 r·min^–1^ for 3 min, absorbed the supernatant, passed this through a 0.22 *μ*m nylon filter membrane to an injection bottle, and waited for sample analysis by the GC-Orbitrap-MS.
2. Sin-QuEChERS Nano column purification: we tested the purification efficiency of the Sin-QuEChERS Nano column, including 2 g Na_2_SO_4_, 0.6 g MgSO_4_, 90 mg PSA, 10 mg C18, and 15 mg MWCNTs. The purification column of the Sin-QuEChERS Nano purification tube is vertically inserted into the 50 ml centrifuge tube containing the extract, and the top of the purification column is slowly pressed down so that the upper organic extract in the centrifuge tube passes through the water blocking filter and column filler in the purification column from bottom to top, and finally enters into the Sin-QuEChERS Nano storage tank for about 4 ml of supernatant. After mixing the purified liquid, the supernatant is sucked over a 0.22 *μ*m nylon filter membrane to the injection bottle for analysis by GC-Orbitrap-MS.

By comparing the purification effects, total ions, and recovery rates of these three purification methods, the Sin-QuEChERS Nano column was finally selected as the purification method for method validation and real sample analysis. See Section 3.2 “Selection of Purification Conditions” for the comparison results.

### 2.4. Preparation of Standard Solution

The mixed standard stock solutions of 352 pesticides were diluted with the blank extract of the matrix, and a series of standard solutions with concentrations of 0.005, 0.02, 0.05, 0.1, and 0.2 *μ*g·ml^−1^ were prepared. The matrix mixed standard solution was prepared and used immediately.

### 2.5. Instrument Conditions

We followed and optimized the methods of previous works [21, 22]. A GC-Orbitrap-MS system (Thermo Scientific, Bremen, Germany) consisting of an AI/AS 1310 TriPlus RSH™ autosampler was used. TRACE 1300 Series GC with a hot split/splitless injector, an EI source, and a hybrid quadrupole Orbitrap mass spectrometer with an HCD (higher energy collision-induced dissociation) cell was used.

GC separation was performed on a 30 m × 0.25 mm id, 0.25 *μ*m Thermo Scientific TG-5MS column using the following temperature program: 40°C, 1.5 min; 25°C·min^−1^ to 90°C, 1.5 min; 25°C·min^−1^ to 180°C, 0 min; 5°C·min^−1^ to 280°C, 0 min; and 10°C·min^−1^ to 310°C, 3 min. Helium 5.0 (99.999%; Linde Gas, Schiedam, The Netherlands) was used as carrier gas at a constant flow of 1 mL·min^−1^. The transfer line was maintained at 280°C. EI was performed at 70 eV, with the source temperature set at 280°C. FS MS acquisition was done in profile mode using an m/z range of 50–550. The nitrogen gas supply for the C-trap was 5.0 grade (99.999%; Linde Gas). The resolving power was set at 60,000 (FWHM at *m*/*z* 200) to ensure high mass accuracy. The automatic gain control (AGC) target was set at 5e^6^ ions, with the maximum ion injection time set to 25 ms.

### 2.6. Establishment of Database

In this experiment, 352 pesticide compounds were selected and prepared into 1.0 *μ*g·ml^−1^ mixed standard solutions. The retention time of the corresponding compounds, the accurate molecular weight, and the chemical formula of the fragment ions were obtained under the full-scan mode. Three fragment ions of each compound were selected to obtain ion information (accurate mass and chemical formula). The data were imported into TraceFinder (4.1) software, and the relevant database was established. The TraceFinder software not only can realize the rapid batch and automatic processing of data but also can set the functions of qualitative, quantitative, and method establishment. According to the established database, it can realize the rapid screening of target substances. The database mainly contains the compounds' names, CAS registration numbers, fragment ion information, retention times, and other information (Table 1).

## 3. Results and Discussion

### 3.1. Optimization of Extraction Conditions

According to the list of pesticides involved in the 2020 edition of Chinese Pharmacopoeia, combined with pesticides, herbicides, and fungicides that may be used in chrysanthemum flower planting, 352 pesticides were selected as the target analytical compounds. Because it contains many pesticides, including organophosphorus, organochlorine, pyrethroids, triazoles, carbamates, and other insecticides, there are many kinds and polarity differences. At the same time, chrysanthemum flower contains pigments, amino acids, and volatile components, so it is particularly important to choose the appropriate extraction solvent. The QuEChERS method uses acetonitrile as the extraction solvent, which is due to the good solubility, permeability, and versatility of acetonitrile and high extraction efficiency for most pesticides. The results showed that the recovery rate of some pesticides with poor stability was low by adding ordinary salt, which was related to the pH value of the matrix; the recovery of 280 pesticides was between 70% and 120%; 38 pesticides were less than 70%; and 34 pesticides were more than 120%. Because carbamates are sensitive to pH value, they are more stable under acidic conditions and easily to decompose under alkaline conditions. Therefore, adding acetate buffer salt makes the sample extract weak acidic, thus improving the recovery rate of acid-base-sensitive pesticides. The recovery rate of all pesticides is between 70% and 125%.

Using the QuEChERS method, adding the appropriate amount of water is conducive to the full contact between organic solvent and sample, improves the extraction efficiency, and helps achieve better recovery. However, adding too much water will lead to the dissolution of water-soluble pigment and other soluble matrix components. The effects of 0, 10, and 15 ml of water on the recovery of the target were compared, and the extraction efficiency of 10 ml water was higher than that of the other two groups. There were only 34 pesticides with a recovery rate of more than 120% in the nonwater group. Therefore, in this method, 10 ml water was added.

Some organophosphorus pesticides (such as parathion and fenitrothion) are unstable in chemical properties and easy to decompose at high temperatures. Because there is anhydrous MgSO_4_ in the acetic acid buffer salt system, a lot of heat will be released in the process of water absorption. Therefore, after adding acetic acid buffer salt, we put the centrifuge tube of extracting sample into an ice water bath for 10 min to improve the recovery rate of pesticides with poor thermal stability.

### 3.2. Selection of Purification Conditions

**Figure 1:**
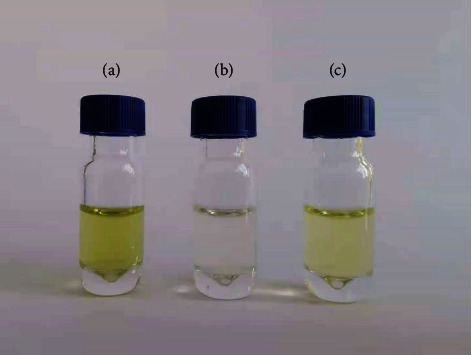
Purification effect maps of chrysanthemum flower samples cleaned up by different purification conditions. (a) QuEChERS purification (simple matrix), (b) QuEChERS purification (complex matrix), and (c) Sin-QuChERS Nano column.

**Figure 2:**
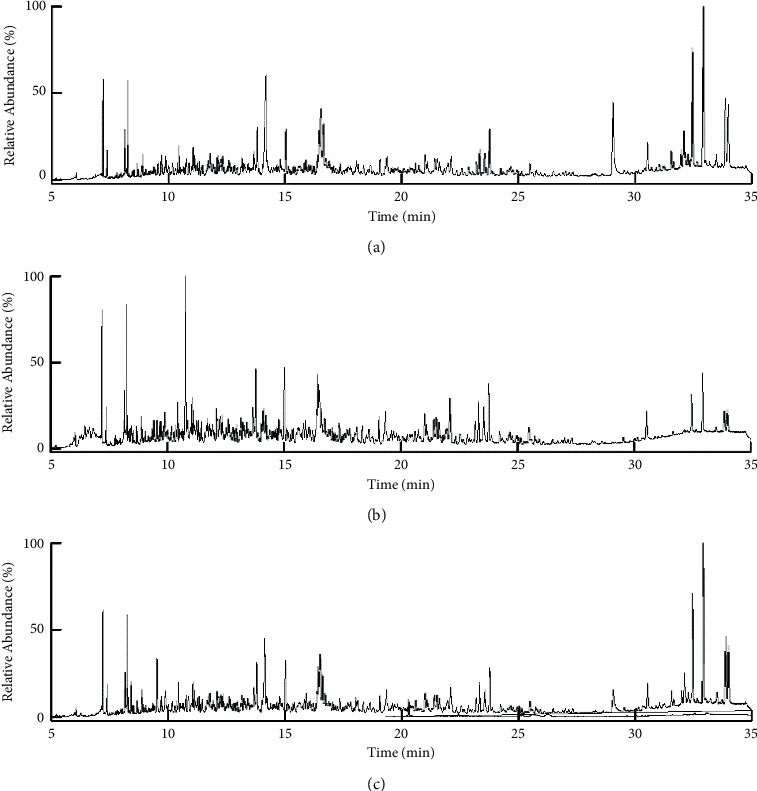
Total ion current chromatograms of the spiked (10 *μ*g ·kg^−1^) chrysanthemum flower samples cleaned up by different purification conditions. (a) QuEChERS purification (simple matrix), (b) QuEChERS purification (complex matrix), and (c) Sin-QuChERS Nano column.

It is important to select suitable purification adsorption materials for the efficient purification of complex substrates. The ideal purification adsorption material should achieve the purification effect required by the experiment and ensure that it does not adsorb the target analyte in the extraction solvent. In this experiment, the purification effects of QuEChERS purification and Sin-QuEChERS Nano column were compared (Figures 1 and 2). Mixed reference materials (10 *μ*g·kg^−1^) were added to the chrysanthemum flower sample and then extracted. The extracts were purified by QuEChERS purification (simple matrix), QuEChERS purification (complex matrix), and Sin-QuEChERS Nano column. It can be seen from Figure 1 that the color of samples purified by QuEChERS purification (simple matrix) is dark, the color of samples purified by Sin-QuEChERS Nano column is lighter, and QuEChERS purification (complex matrix) is almost colorless. It can be seen from the total ions in Figure 2 that the samples purified by QuEChERS purification (simple matrix) have more impurities and greater interference, while the samples purified by the Sin-QuEChERS Nano column are less interfered with, and the peak of QuEChERS purification (complex matrix) is less after 25 min, which is due to the adsorption of the target substance with late peak, making it look cleaner. Meanwhile, the recovery rates of target compounds were 72.7–118.9% in QuEChERS purification (simple matrix), 72.8–123.4% with the Sin-QuEChERS Nano column, and 62.4–120.7% by QuEChERS purification (complex matrix). The results showed little difference in the recovery rate between QuEChERS purification (simple matrix) and Sin-QuEChERS Nano column, but the recovery rate of QuEChERS purification (complex matrix) was relatively low. Primary-secondary amine (PSA), which plays the main role in QuEChERS purification (simple matrix), is a weak anion exchange adsorbent. It can effectively remove polar pigments, organic acids, sugars, fatty acids, and other components that are easy to form hydrogen bonds in the sample, but its adsorption capacity is limited. In addition to PSA, QuEChERS purification (complex matrix) also contains graphitized carbon black (GCB). GCB can remove pigments from chrysanthemum flower, such as chlorophyll, radish-like hormone, and sterol, but the strong adsorption force will absorb the target of a benzene ring, which leads to a low recovery rate. In addition to PSA, 15 mg MWCNTs (particle size length: 10–50 *μ*m, outer diameter: 30–60 nm, and specific surface area: 280 m^2^·g^−1^) was added to the Sin-QuEChERS Nano column. MWCNTs are nano hollow tubes with high mechanical strength, strong acid-base resistance, stronger adsorption, and purification capacity but do not affect the recovery rate of the target substance [23–25]. This experiment shows that the combination of PSA and MWCNTs can effectively remove impurities in the sample, reduce the interference to the target substance, improve the recovery rate of the target substance, and protect the analytical instrument from pollution and damage. At the same time, the high-resolution mass spectrometer can detect low concentration pesticide residues in a complex matrix, so the Sin-QuEChERS Nano column was selected for purification.

### 3.3. Optimization of Instrument Resolution

**Figure 3:**
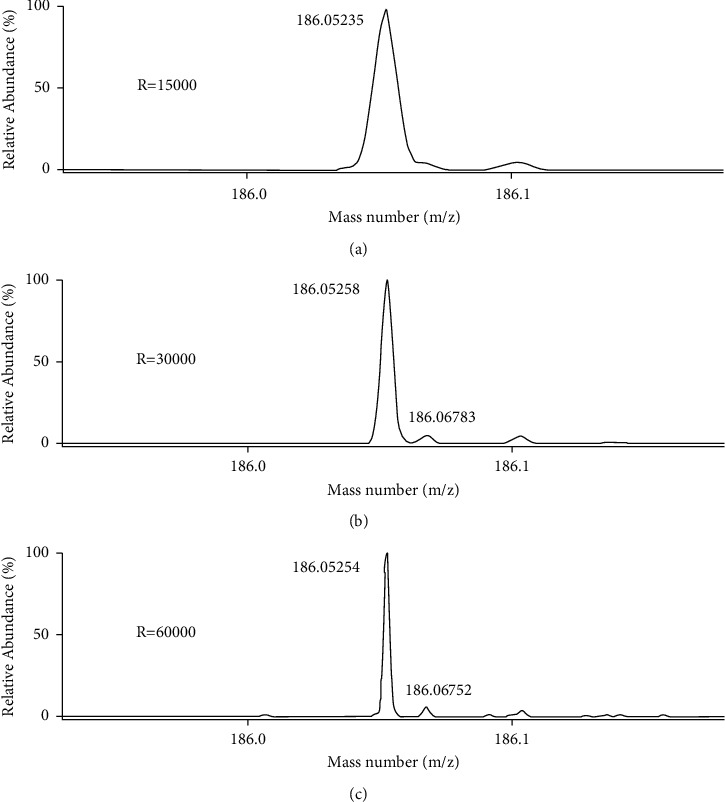
Effect of different resolutions ((a) 15,000, (b) 30,000, and (c) 60,000) on the quality accuracy of the qualitative ion (*m*/*z* 186.05251) of trifloxystrobin in chrysanthemum flower.

As a high-resolution mass spectrometer, Orbitrap mass spectrometer can fully scan acquisition and collect data in the range of *m*/*z* 50–550, ensuring the retrospective data analysis. Resolution is an important parameter in high-resolution mass spectrometry. In the presence of matrix interference, the resolution will affect the accuracy of quality measurement. Therefore, the key to qualitative analysis is to choose the appropriate resolution. High resolution can improve the accuracy of mass determination and can effectively identify compounds with very close accurate mass. In the experiment, the content of trifloxystrobin in chrysanthemum flower was 10 *μ*g ·kg^−1^, which was determined at three different resolutions (15,000, 30,000, and 60,000). In Figure 3, the qualitative ion *m*/*z* 186.05251 is the qualitative ion of trifloxystrobin, and *m*/*z* 186.06752 is the interference ion. Only when the resolution is 60,000 or above, the two ions with the same mass can be clearly distinguished. At the same time, the accurate qualitative and quantitative analysis can be carried out, and the screening accuracy will be greatly improved; the quality accuracy is less than 2.0 ppm; and the high sensitivity can still be maintained, so it fully meets the requirements of pesticide residue detection in chrysanthemum flower. Also, the accurate mass number and deviation, retention time window, isotopic distribution, and isotopic abundance information were used simultaneously in this method to realize the rapid and accurate screening of target substances.

### 3.4. Matrix Effect

Matrix effects (MEs) are very common in GC-MS/MS and should be assessed at the method validation stage. MEs were estimated via the ratio of the calibration curve slopes of matrix to solvent. Studies recommend that MEs can be ignored when the ME values are in the range of 0.9–1.1 [15]. If the ME cannot be ignored, using a matrix-matched standard is the most effective way to compensate for MEs.

The MEs in this study are listed in Table S1. The MEs of the Sin-QuEChERS Nano method were in the range of 1.01–1.86; the MEs of the QuEChERS (simple matrix) and QuEChERS (complex matrix) method ranged between 1.05 and 2.38 and between 1.08 and 2.89, respectively. As for the matrix suppression or enhancement effect, QuEChERS (simple matrix) was the strongest, while Sin-QuEChERS nano was the weakest. This indicated that the Sin-QuEChERS Nano method reduced the matrix effect more efficiently than QuEChERS (simple matrix) and QuEChERS (complex matrix).

### 3.5. Linear Range, Limit of Detection, Limit of Quantitation, and Recovery

The blank matrix standard solution is prepared according to the pretreatment method; the standard curve was drawn with the mass concentration of the compound as abscissa and the corresponding peak area as ordinate. The linear range of 352 compounds was 0.5–200 *μ*g ·kg^−1^, and the correlation coefficient (R) was greater than 0.99. The LODs and LOQs of the method were investigated by adding blank samples. The LODs were three times the signal-to-noise ratio (*S*/*N* = 3), and the LOQs were 10 times the signal-to-noise ratio (*S*/*N* = 10). The mixed standard solutions of 352 compounds were added to the negative samples of chrysanthemum flower at the levels of 10, 50, and 100 *μ*g·kg^−1^, respectively, and each level was repeated six times. The results showed that the detection limits of 352 pesticides were 0.3–3 *μ*g·kg^−1^, and the quantification limits were 1–10.0 *μ*g·kg^−1^, which met the requirements of pesticide residue detection [26]. The average recovery rates of 352 compounds at three levels were 73.2–110.3%, 72.8–112.6%, and 77.6–123.4%, respectively. The average RSDs of 352 compounds at three levels were 3.2–9.6%, 4.0–9.7%, and 4.0–11.3%, respectively. The results showed that the method could be used for the determination of pesticide residues in chrysanthemum flower. The correlation coefficients, limits of detection, limits of quantitation, spiked recovery rates, and relative standard deviations of 352 compounds in chrysanthemum flower are shown in Table S1.

### 3.6. Determination of Actual Samples

**Figure 4:**
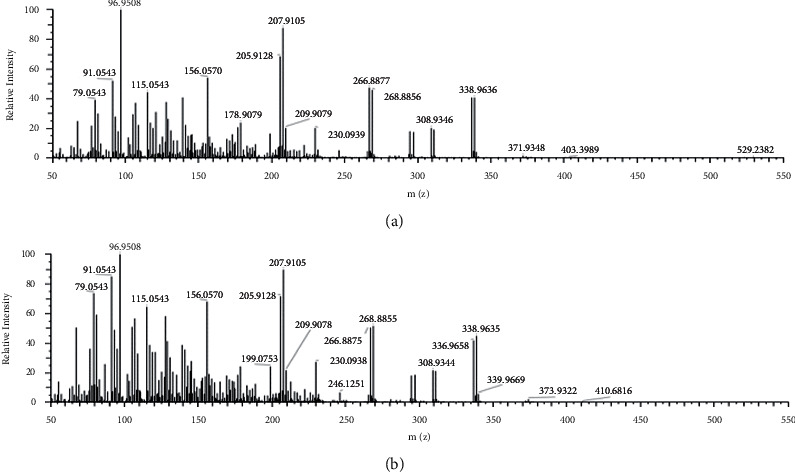
Mass spectrum of profenofos in the standard (a) and chrysanthemum flower positive samples (b).

Two hundred samples were analyzed by the established method. Among them, 137 samples were detected with pesticide residues, and the chemical substances with a high detection rate were profenofos, procymidone, metalaxyl, chlorfenapyr, difenoconazole, dimethomorph, cypermethrin, tebuconazole, propiconazole, and pyrimethanil, among others (Table 2).  Figure 4 is the mass spectrum of profenofos in the standard and chrysanthemum flower positive samples. The fragment ions (338.96369, 205.91286, and 207.91063) can be detected, and the ion ratio is highly matched. The results show that the method was also suitable for detecting 352 pesticide residues in chrysanthemum flowers, such as calendula and chamomile. The results showed that the nontarget rapid screening method established in this study could rapidly screen potential pesticide residues in chrysanthemum flower with high throughput.

### 3.7. Retrospective Analysis

GC-Orbitrap-MS often collects the full spectrum, which can collect data more comprehensively. The data collection has no relationship with the number of compounds in the database, so the data can be reviewed and reanalyzed to expand the target range. In the analysis of samples, we added the retention time, molecular formula, accurate relative molecular weight, and CAS number of new compounds pentachlorobenzonitrile, simazine, and simetone into 352 databases and verified them with actual samples. It was found that the linearity of these three compounds in each matrix was greater than 0.99, the average recovery rates were 72.8–123.4%, and the average RSD values were 3.2–11.3% at three levels (10, 50, and 100 *μ*g·kg^−1^, respectively), which met the requirements of detection. Among 200 chrysanthemum flower samples, 5 chrysanthemum flower samples were detected with pentachlorobenzonitrile with a detection value range of 0.048–0.22 mg·kg^−1^; 3 chrysanthemum flower samples were detected with simetone, with a detection value range of 0.032–0.051 mg·kg^−1^; and no samples were detected with simazine. Retrospective analyses can expand and analyze target compounds without recollecting data, which is flexible and is convenient for high-throughput screening and quantitative analysis of pesticide residues. It is the development direction of chrysanthemum flower risk monitoring technology in the future.

## 4. Conclusion

The work presented a method that had been developed and validated for the simultaneous determination of 352 pesticide residues in chrysanthemum flower by GC-Orbitrap-MS, which was established based on the purification of the Sin-QuEChERS Nano column. The Sin-QuEChERS Nano column simplifies the pretreatment process and effectively improves the purification efficiency. After systematic validation for linearity, precision, accuracy, stability, and matrix effects, the developed method was successfully applied for qualitative confirmation and quantitative detection of 352 pesticide residues in 200 chrysanthemum flower samples bought from local pharmacies. No saturation phenomena were experienced in any case. The developed and validated method has proved to be robust and appropriate in sensitivity, mass accuracy, and quantification in full-scan mode and provide good results in the analysis of real samples. These good results show the advantages of full-scan analysis, which is applicable to other compounds that do not appear in selective and retrospective evaluation and easier range management than GC-MS/MS. This method has the advantages of simple pretreatment, high purification efficiency, high throughput, and accurate analysis. It can effectively reduce the amount of standard substances in the detection of multipesticide residues in chrysanthemum flowers, which provides technical support for rapid screening and analysis of potential pesticide residues in the chrysanthemum flower.

## Figures and Tables

**Table 1:** Information of the 352 pesticides detected in chrysanthemum flower samples by GC-Orbitrap-MS screening.

Pesticides	CAS	Molecular formula	Retention time (min)	Quantitative ion (*m*/*z*)	Qualitative ion (*m*/*z*)	
					1	2
Clopyralid	1702-17-6	C_6_H_3_C_l2_NO_2_	6.38	146.96	76.02	111.99
Dichlorvos	62-73-7	C_4_H_7_O_4_PC_l2_	7.99	184.98	186.97	144.98
Methamidophos	10265-92-6	C_2_H_8_NO_2_PS	8.02	141.00	112.01585	125.98
Thiofanox	39196-18-4	C_9_H_18_N_2_O_2_S	8.20	115.10	161.09	83.07
Allidochlor	93-71-0	C_8_H_12_NOCl	8.27	138.09	132.02	96.08
Dichlobenil	1194-65-6	C_7_H_3_C_l2_N	8.68	170.96	100.02	172.96
EPTC	759-94-4	C_9_H_19_NOS	8.71	128.11	132.08	160.08
Dichlormid	37764-25-3	C_8_H_11_C_l2_NO	8.72	172.05	108.08	165.98
2,4,6-Trichlorophenol	88-06-2	C_6_H_3_OCl_3_	8.73	195.92	199.92	197.92
3,5-Dichloroaniline	626-43-7	C_6_H_5_C_l2_N	9.05	160.98	162.98	126.01
O-phthalimide	85-41-6	C_8_H_5_NO_2_	9.15	147.03	103.04	104.03
Mevinphos	7786-34-7	C_7_H_13_O_6_P	9.18	192.02	164.02	127.01
Acephate	30560-19-1	C_4_H_10_NO_3_PS	9.25	136.02	112.02	94.00
Vernolate	1929-77-7	C_10_H_21_NOS	9.30	86.06	161.09	146.10
Propham	122-42-9	C_10_H_13_NO_2_	9.37	137.05	179.09	120.08
Etridiazole	2593-15-9	C_5_H_5_N_2_OSC_l3_	9.40	210.95	212.95	182.92
Pebulate	1114-71-2	C_10_H_21_NOS	9.40	128.11	72.04	161.09
cis-1,2,3,6-Tetrahydrophthalimide	1469-48-3	C_8_H_9_NO_2_	9.57	151.06	123.07	122.06
Chloroneb	2675-77-6	C_8_H_8_O_2_C_l2_	9.77	190.97	192.96	205.99
Tebuthiuron	34014-18-1	C_9_H_16_N_4_OS	9.86	156.06	89.02	171.08
Fenobucarb	3766-81-2	C_12_H_17_NO_2_	9.93	121.06	91.05	93.07
Pentachlorobenzene	608-93-5	C_6_HC_l5_	9.95	247.85	251.85	249.85
Isoprocarb	2631-40-5	C_11_H_15_NO_2_	9.98	121.06	136.09	103.05
Molinate	2212-67-1	C_9_H_17_NOS	10.01	126.09	187.10	98.10
Heptenophos	23560-59-0	C_9_H_12_CLO_4_P	10.30	124.00	215.05	200.02
Chlorfenprop-methyl	14437-17-3	C_10_H_10_O_2_Cl_2_	10.43	165.01	196.03	167.00
Omethoate	1113-02-6	C_5_H_12_NO_4_PS	10.47	156.00	110.01	140.98
Propoxur	114-26-1	C_11_H_15_NO_3_	10.57	110.04	82.04	152.08
Tecnazene	117-18-0	C_6_HNO_2_CL_4_	10.59	200.88	177.91	260.87
Propachlor	1918-16-7	C_11_H_14_NOCl	10.61	120.08	176.11	169.03
Diphenylamine	122-39-4	C_12_H_11_N	10.70	169.09	167.07	168.08
Ethoprophos	13194-48-4	C_8_H_19_O_2_PS_2_	10.77	157.96	199.00	200.01
Tributyl phosphate	126-73-8	C_12_H_27_O_4_P	10.78	98.98	155.05	124.10
Cycloate	1134-23-2	C_11_H_21_NOS	10.79	154.12	155.13	72.04
2,3,5,6-Tetrachloroaniline	3481-20-7	C_6_H_3_Cl_4_N	10.80	230.90	232.90	157.96
Atrazine-desethyl	6190-65-4	C_6_H_10_N_5_Cl	11.00	172.04	145.01	187.06
Dicrotophos	141-66-2	C_8_H_16_NO_5_P	11.07	127.02	193.03	111.07
Methabenzthiazuron	18691-97-9	C_10_H_11_N_3_OS	11.07	136.02	164.04	135.01
Triﬂuralin	1582-09-8	C_13_H_16_N_3_O_4_F_3_	11.08	264.02	306.07	248.03
Bendiocarb	22781-23-3	C_11_H_13_NO_4_	11.09	151.04	126.03	223.08
Benﬂuralin	1861-40-1	C_13_H_16_F_3_N_3_O_4_	11.12	292.05	276.06	318.11
Sulfotep	3689-24-5	C_8_H_20_O_5_P_2_S_2_	11.20	322.02	209.90	173.96
Cadusafos	95465-99-9	C_10_H_23_O_2_PS_2_	11.25	158.97	130.94	213.02
Tebutam	35256-85-0	C_15_H_23_NO	11.27	91.05	190.12	233.18
Promecarb	2631-37-0	C_12_H_17_NO_2_	11.27	135.08	107.09	150.10
Phorate	298-02-2	C_7_H_17_O_2_PS_3_	11.34	75.03	230.97	260.01
*α*-Hexachlorocyclohexane	319-84-6	C_6_H_6_Cl_6_	11.95	180.94	145.97	218.91
Atratone	1610-17-9	C_9_H_17_N_5_O	11.61	169.10	154.07	211.14
3,4,5-Trimethacarb	2686-99-9	C_11_H_15_NO_2_	11.69	121.06	91.05	136.09
Dicloran	99-30-9	C_6_H_4_Cl_2_N_2_O_2_	11.70	175.97	159.97	207.96
Pentachloroanisole	1825-21-4	C_7_H_3_Cl_5_O	11.72	264.84	238.84	279.86
Ethoxyquin	91-53-2	C_14_H_19_NO	11.72	202.12	174.09	203.13
Prometon	1610-18-0	C_10_H_19_N_5_O	11.73	168.09	210.13	225.16
Atrazine	1912-24-9	C_8_H_14_ClN_5_	11.84	200.07	202.07	173.05
Monolinuron	1746-81-2	C_9_H_11_ClN_2_O_2_	11.89	126.01	152.10	214.05
Propazine	139-40-2	C_9_H_16_N_5_Cl	11.92	214.08	187.06	229.11
Clomazone	81777-89-1	C_12_H_14_ClNO_2_	11.94	204.10	89.04	125.02
Terbumeton	33693-04-8	C_10_H_19_N_5_O	11.95	210.13	169.10	225.16
*β*-Hexachlorocyclohexane	319-85-7	C_6_H_6_Cl_6_	11.99	180.94	145.97	218.91
Aminocarb	2032-59-9	C_11_H_16_N_2_O_2_	12.04	151.10	136.08	150.09
Isocarbamid	30979-48-7	C_8_H_15_N_3_O_2_	12.05	142.06	130.06	113.03
Cyromazine	66215-27-8	C_6_H_10_N_6_	12.06	151.07	165.09	166.10
*γ*-Hexachlorocyclohexane	58-89-9	C_6_H_6_Cl_6_	12.12	180.94	145.97	218.91
Propetamphos	31218-83-4	C_10_H_20_NO_4_PS	12.12	138.01	193.98	222.03
Cycluron	2163-69-1	C_11_H_22_N_2_O	12.13	198.17	127.09	169.13
Terbuthylazine	5915-41-3	C_9_H_16_N_5_Cl	12.14	186.05	188.05	201.08
Terbufos	13071-79-9	C_9_H_21_O_2_PS_3_	12.16	230.97	174.91	202.94
Cyanophos	2636-26-2	C_9_H_10_NO_3_PS	12.17	243.01	124.98	109.00
Trietazine	1912-26-1	C_9_H_16_N_5_Cl	12.17	200.07	214.09	229.11
Quintozene	82-68-8	C_6_NO_2_Cl_5_	12.23	213.87	248.84	294.83
Fonofos	944-22-9	C_10_H_15_OPS_2_	12.27	246.03	137.02	108.99
Pyroquilon	57369-32-1	C_11_H_11_NO	12.28	173.08	144.08	172.08
Dinoterb	1420-07-1	C_10_H_12_N_2_O_5_	12.35	225.05	177.04	161.05
Pyrimethanil	53112-28-0	C_12_H_13_N_3_	12.35	198.10	199.11	183.08
Diazinon	333-41-5	C_12_H_21_N_2_O_3_PS	12.36	179.12	199.06	304.10
Flufenoxuron	101463-69-8	C_21_H_11_CLF_6_N_2_O_3_	12.46	331.00	268.04	296.03
Disulfoton	298-04-4	C_8_H_19_O_2_PS_3_	12.51	88.03	153.01	141.97
Paraoxon-methyl	950-35-6	C_8_H_10_NO_6_P	12.52	230.02	247.02	200.02
Secbumeton	26259-45-0	C_10_H_19_N_5_O	12.52	196.12	169.10	210.13
Aziprotryne	4658-28-0	C_7_H_11_N_7_S	12.53	182.05	139.01	225.08
Dinitramine	29091-05-2	C_11_H_13_F_3_N_4_O_4_	12.53	305.09	244.06	261.06
Fenfuram	24691-80-3	C_12_H_11_NO_2_	12.57	201.08	184.05	109.03
*δ*-Hexachlorocyclohexane	319-86-8	C_6_H_6_Cl_6_	12.61	180.94	145.97	218.91
Mexacarbate	315-18-4	C_12_H_18_N_2_O_2_	12.63	165.11	164.11	222.14
Isazofos	42509-80-8	C_7_H_13_N_3_O_3_PSCl	12.66	162.04	161.03	177.01
Chlorothalonil	1897-45-6	C_8_Cl_4_N_2_	12.71	263.88	193.94	228.91
Triallate	2303-17-5	C_10_H_16_CL_3_NOS	12.71	268.03	270.03	142.92
Tebupirimfos	96182-53-5	C_13_H_23_N_2_O_3_PS	12.82	234.02	261.05	276.07
Musk ambrette	83-66-9	C_12_H_16_N_2_O_5_	12.83	253.08	251.10	268.10
Oxabetrinil	74782-23-3	C_12_H_12_N_2_O_3_	12.85	73.03	103.04	114.03
Iprobenfos	26087-47-8	C_13_H_21_O_3_PS	12.87	204.00	171.02	246.05
Fluroxypyr	69377-81-7	C_7_H_5_N_2_O_3_FCl_2_	12.92	180.97	208.97	195.96
Pirimicarb	23103-98-2	C_11_H_18_N_4_O_2_	12.95	238.14	166.10	137.07
Monalide	7287-36-7	C_13_H_18_NOCl	12.95	197.06	127.01	239.11
Furmecyclox	60568-05-0	C_14_H_21_NO_3_	12.98	123.04	251.15	124.05
Benoxacor	98730-04-2	C_11_H_11_NO_2_C_l2_	12.99	120.04	259.02	261.01
Pentachloroaniline	527-20-8	C_6_H_2_C_l5_N	13.14	262.86	191.92	229.89
Benfuresate	68505-69-1	C_12_H_16_O_4_S	13.17	163.08	121.06	256.08
Dioxacarb	6988-21-2	C_11_H_13_NO_4_	13.17	121.03	165.05	166.06
Cyprazine	22936-86-3	C_9_H_14_N_5_Cl	13.24	212.07	170.02	226.08
Phosphamidon	13171-21-6	C_10_H_19_NO_5_PCl	13.25	138.09	193.02	264.10
Dichlorprop	120-36-5	C_9_H_8_O_3_Cl_2_	13.26	161.96	132.96	188.99
Dichlofenthion	97-17-6	C_10_H_13_CL_2_O_3_PS	13.26	222.94	250.97	279.00
Fenthion	55-38-9	C_10_H_15_O_3_PS_2_	13.26	222.94	250.97	279.00
Propanil	709-98-8	C_9_H_9_NOCl_2_	13.26	160.98	162.98	219.00
2,4-DB	94-82-6	C_10_H_10_O_3_C_l2_	13.26	161.96	125.99	97.99
Chlorthiamid	1918-13-4	C_7_H_5_Cl_2_NS	13.29	169.98	171.98	204.95
Dimethachlor	50563-36-5	C_13_H_18_NO_2_Cl	13.29	197.06	148.08	134.10
Metribuzin	21087-64-9	C_8_H_14_N_4_OS	13.31	198.07	144.05	182.04
Dimethenamid	87674-68-8	C_12_H_18_NO_2_SCl	13.32	154.07	230.04	232.04
Bromobutide	74712-19-9	C_15_H_22_NOBr	13.35	119.09	120.08	232.17
Terbucarb	1918-11-2	C_17_H_27_NO_2_	13.44	205.16	177.13	220.18
Malaoxon	1634-78-2	C_10_H_19_O_7_PS	13.46	268.02	194.99	238.98
Vinclozolin	50471-44-8	C_12_H_9_Cl_2_NO_3_	13.48	178.04	212.00	285.10
Parathion-methyl	298-00-0	C_8_H_10_NO_5_PS	13.49	263.00	124.98	245.99
Chlorpyrifos-methyl	5598-13-0	C_7_H_7_Cl_3_NO_3_PS	13.50	285.93	287.92	289.92
Transfluthrin	118712-89-3	C_15_H_12_O_2_F_4_Cl_2_	13.54	163.02	127.03	335.05
Simetryn	1014-70-6	C_8_H_15_N_5_S	13.56	213.10	155.04	170.05
Fuberidazole	3878-19-1	C_11_H_8_N_2_O	13.57	184.06	156.07	183.06
Tolclofos-methyl	57018-04-9	C_9_H_11_O_3_PSCL_2_	13.61	264.98	249.96	266.98
Alachlor	15972-60-8	C_14_H_20_NO_2_Cl	13.67	188.11	202.12	160.11
Ametryn	834-12-8	C_9_H_17_N_5_S	13.67	227.12	170.05	185.07
Heptachlor	76-44-8	C_10_H_5_CL_7_	13.72	269.81	100.01	336.85
Prometryn	7287-19-6	C_10_H_19_N_5_S	13.75	241.13	184.07	199.09
Acetochlor	34256-82-1	C_14_H_20_NO_2_Cl	13.76	223.08	162.10	174.10
Paraoxon-ethyl	311-45-5	C_10_H_14_NO_6_P	13.77	275.05	247.02	139.05
Metalaxyl	57837-19-1	C_15_H_21_NO_4_	13.78	160.11	206.12	146.10
Tridiphane	58138-08-2	C_10_H_7_OC_l5_	13.84	186.97	172.96	284.92
Octachlorodipropyl ether	127-90-2	C_6_H_6_OCl_8_	13.89	129.91	108.96	142.92
Prosulfocarb	52888-80-9	C_14_H_21_NOS	13.89	128.11	86.06	251.13
Fenpropidin	67306-00-7	C_19_H_31_N	13.97	98.10	273.24	258.22
1-naphthylacetamide	86-86-2	C_12_H_11_NO	14.05	141.07	142.08	185.08
Dithiopyr	97886-45-8	C_15_H_16_NO_2_F_5_S_2_	14.05	306.05	258.05	354.06
Orbencarb	34622-58-7	C_12_H_16_NOSCl	14.08	222.09	125.02	100.08
Terbutryn	886-50-0	C_10_H_19_N_5_S	14.08	226.11	185.07	170.05
Spiroxamine	118134-30-8	C_18_H_35_NO_2_	14.11	100.11	126.13	198.15
Methiocarb	2032-65-7	C_11_H_15_NO_2_S	14.15	168.06	153.04	154.04
Fenitrothion	122-14-5	C_9_H_12_NO_5_PS	14.16	260.01	124.98	277.02
Pirimiphos-methyl	29232-93-7	C_11_H_20_N_3_O_3_PS	14.19	290.07	276.06	305.10
Methiocarb sulfone	2179-25-1	C_11_H_15_NO_4_S	14.22	200.05	197.03	197.03
Ethofumesate	26225-79-6	C_13_H_18_O_5_S	14.22	207.10	161.06	179.07
Linuron	330-55-2	C_9_H_10_N_2_O_2_Cl_2_	14.27	159.97	61.05	248.01
Probenazole	27605-76-1	C_10_H_9_NO_3_S	14.33	130.07	103.04	158.06
Noruron	18530-56-8	C_13_H_22_N_2_O	14.34	153.10	193.13	207.15
Quinoclamine	2797-51-5	C_10_H_6_NO_2_Cl	14.37	172.04	144.04	207.01
Dipropetryn	4147-51-7	C_11_H_21_N_5_S	14.38	255.15	222.17	184.07
Malathion	121-75-5	C_10_H_19_O_6_PS_2_	14.41	124.98	99.01	173.08
Thiobencarb	28249-77-6	C_12_H_16_ClNOS	14.44	257.06	100.08	125.02
Diethofencarb	87130-20-9	C_14_H_21_NO_4_	14.51	267.15	225.10	168.03
Phorate sulfoxide	2588-03-6	C_7_H_17_O_3_PS_3_	14.57	124.93	170.97	199.00
Metolachlor	51218-45-2	C_15_H_22_NO_2_Cl	14.61	162.13	211.08	238.10
Fenpropimorph	67564-91-4	C_20_H_33_NO	14.67	128.11	110.10	173.13
Cyanazine	21725-46-2	C_9_H_13_ClN_6_	14.69	225.07	212.06	240.09
Chlorpyrifos	2921-88-2	C_9_H_11_CL_3_NO_3_PS	14.71	196.92	257.90	313.96
Parathion	56-38-2	C_10_H_14_NO_5_PS	14.73	291.03	155.00	185.99
Flufenacet	142459-58-3	C_14_H_13_N_3_O_2_F_4_S	14.79	210.98	136.06	151.08
Rabenzazol	40341-04-6	C_12_H_12_N_4_	14.79	212.11	170.07	195.08
4,4′-Dichlorobenzophenone	90-98-2	C_13_H_8_Cl_2_O	14.80	138.99	110.99	249.99
Triadimefon	43121-43-3	C_14_H_16_ClN_3_O_2_	14.80	208.03	210.02	181.02
Chlorthal-dimethyl	1861-32-1	C_10_H_6_CL_4_O_4_	14.86	300.88	298.88	331.90
Dicapthon	2463-84-5	C_8_H_9_NO_5_PSCl	14.86	261.99	124.98	216.00
Isofenphos-oxon	31120-85-1	C_15_H_24_NO_5_P	14.87	200.99	229.03	272.07
Isocarbophos	24353-61-5	C_11_H_16_NO_4_PS	14.90	135.99	230.00	121.03
Tetraconazole	112281-77-3	C_13_H_11_Cl_2_F_4_N_3_O	14.92	336.05	136.01	170.98
Isobenzan	297-78-9	C_9_H_4_CL_8_O	15.00	407.78	274.86	310.83
Flurochloridone	61213-25-0	C_12_H_10_Cl_2_F_3_NO	15.01	174.05	311.01	313.01
Fenson	80-38-6	C_12_H_9_O_3_SCl	15.03	267.99	141.00	269.99
Pyracarbolid	24691-76-7	C_13_H_15_NO_2_	15.13	125.06	217.11	97.03
Dodemorph	1593-77-7	C_18_H_35_NO	15.13	154.12	238.22	281.27
Mgk 264	113-48-4	C_17_H_25_NO_2_	15.14, 15.45	164.07	209.14	210.15
Butralin	33629-47-9	C_14_H_21_N_3_O_4_	15.16	266.11	236.10	220.11
Carbaryl	63-25-2	C_12_H_11_NO_2_	15.16	144.06	115.05	116.06
Diphenamid	957-51-7	C_16_H_17_NO	15.21	167.09	165.07	152.06
Pirimiphos-ethyl	23505-41-1	C_13_H_24_N_3_O_3_PS	15.29	168.06	318.10	333.13
Isodrin	465-73-6	C_12_H_8_C_l6_	15.36	192.94	361.88	194.93
Aldrin	309-00-2	C_12_H_8_CL_6_	15.36	260.86	290.93	326.91
Isopropalin	33820-53-0	C_15_H_23_N_3_O_4_	15.36	280.13	238.08	264.13
Cyprodinil	121552-61-2	C_14_H_15_N_3_	15.41	224.12	225.13	208.09
Isofenphos-methyl	99675-03-3	C_14_H_22_NO_4_PS	15.42	199.02	230.99	241.06
Octachlorostyrene	29082-74-4	C_8_Cl_8_	15.54	305.81	270.84	379.74
Metazachlor	67129-08-2	C_14_H_16_ClN_3_O	15.56	209.06	133.09	211.06
Dimethametryn	22936-75-0	C_11_H_21_N_5_S	15.58	212.10	185.07	240.13
Pendimethalin	40487-42-1	C_13_H_19_N_3_O_4_	15.60	252.10	191.07	162.08
Disulfoton-sulfone	2497-6-5	C_8_H_19_O_4_PS_3_	15.62	153.01	124.98	213.02
Phorate sulfone	2588-04-7	C_7_H_17_O_4_PS_3_	15.62	199.00	124.98	170.97
Terbufos sulfone	56070-16-7	C_9_H_21_O_4_PS_3_	15.62	153.01	199.00	263.97
Paclobutrazol	76738-62-0	C_15_H_20_ClN_3_O	15.64	236.06	138.02	167.03
Penconazole	66246-88-6	C_13_H_15_N_3_Cl_2_	15.64	248.09	160.97	158.98
Chlozolinate	84332-86-5	C_13_H_11_NO_5_Cl_2_	15.72	186.96	260.98	188.96
Pyrifenox	88283-41-4	C_14_H_12_N_2_OCl_2_	15.72	262.01	186.96	227.04
Tolylfluanid	731-27-1	C_10_H_13_N_2_O_2_FS_2_Cl_2_	15.75	237.97	181.08	239.96
Fosthiazate	98886-44-3	C_9_H_18_NO_3_PS_2_	15.80	195.01	166.02	226.98
Phosfolan	947-02-4	C_7_H_14_NO_3_PS_2_	15.80	139.96	167.99	266.98
Allethrin	584-79-2	C_19_H_26_O_3_	15.84	123.12	91.05	136.09
Isofenphos	25311-71-1	C_15_H_24_NO_4_PS	15.84	213.03	184.99	216.97
Captan	133-06-2	C_9_H_8_NO_2_SCl_3_	15.85	149.05	105.03	116.91
Fipronil	120068-37-3	C_12_H_4_Cl_2_F_6_N_4_OS	15.88	366.94	368.94	212.95
Diclocymet	139920-32-4	C_15_H_18_N_2_OCl_2_	15.90, 16.38	221.05	172.99	277.11
Quinalphos	13593-03-8	C_12_H_15_N_2_O_3_PS	15.92	146.05	157.08	173.07
Phenthoate	2597-03-7	C_12_H_17_O_4_PS_2_	15.93	273.99	121.01	245.99
Triadimenol	55219-65-3	C_14_H_18_N_3_O_2_Cl	15.94	168.11	112.05	169.12
Dinobuton	973-21-7	C_14_H_18_N_2_O_7_	16.00	211.03	163.03	205.06
Furalaxyl	57646-30-7	C_17_H_19_NO_4_	16.03	242.12	152.07	146.10
Crotoxyphos	7700-17-6	C_14_H_19_O_6_P	16.06	193.03	127.02	105.07
Procymidone	32809-16-8	C_13_H_11_NO_2_Cl_2_	16.11	283.02	96.06	255.02
Chlorbenside	103-17-3	C_13_H_10_SCl_2_	16.15	125.02	127.01	267.99
Chlorflurenol-methyl	2536-31-4	C_15_H_11_O_3_Cl	16.22	215.03	152.06	274.04
Chlordane	5103-71-9	C_10_H_6_Cl_8_	16.32, 16.58	372.83	376.82	374.82
Methidathion	950-37-8	C_6_H_11_N_2_O_4_PS_3_	16.33	145.01	85.04	147.00
Haloxyfop-methyl	69806-40-2	C_16_H_13_ClF_3_NO_4_	16.39	375.05	288.00	179.98
Bromophos-ethyl	4824-78-6	C_10_H_12_O_3_PSCl_2_Br	16.41	300.85	241.87	358.91
Procyazine	32889-48-8	C_10_H_13_N_6_Cl	16.43	210.05	212.05	252.09
Disulfoton-sulfoxide	2497-07-6	C_8_H_19_O_3_PS_3_	16.61	183.98	124.98	167.98
Tetrachlorvinphos	22248-79-9	C_10_H_9_O_4_PCl_4_	16.63	328.93	203.93	239.89
Endosulfan	959-98-8	C_9_H_6_O_3_SCl_6_	16.67, 18.37	236.84	169.97	159.98
Mepanipyrim	110235-47-7	C_14_H_13_N_3_	16.69	222.10	221.09	223.11
Butachlor	23184-66-9	C_17_H_26_NO_2_Cl	16.74	176.11	188.11	160.11
Ditalimfos	5131-24-8	C_12_H_14_NO_4_PS	16.81	242.98	208.97	271.00
TCMTB	21564-17-0	C_9_H_6_N_2_S_3_	16.87	179.99	166.99	237.97
Trans-nonachlor	39765-80-5	C_10_H_5_Cl_9_	16.90	408.78	404.79	271.81
Chlorfenson	80-33-1	C_12_H_8_CL_2_O_3_S	16.94	174.96	176.96	301.96
Fenamiphos	22224-92-6	C_13_H_22_NO_3_PS	16.95	303.11	260.05	217.01
Picoxystrobin	117428-22-5	C_18_H_16_NO_4_F_3_	16.96	303.05	173.06	335.08
Napropamide	15299-99-7	C_17_H_21_NO_2_	17.00	271.16	72.08	115.05
Hexaconazole	79983-71-4	C_14_H_17_Cl_2_N_3_O	17.06	213.99	231.03	174.97
Flutolanil	66332-96-5	C_17_H_16_NO_2_F_3_	17.07	173.02	281.07	323.11
Prothiophos	34643-46-4	C_11_H_15_O_2_PS_2_Cl_2_	17.18	308.99	238.92	266.95
Isoprothiolane	50512-35-1	C_12_H_18_O_4_S_2_	17.19	117.99	161.98	290.06
Profenofos	41198-08-7	C_11_H_15_BrClO_3_PS	17.26	338.96	205.91	207.91
Tricyclazole	41814-78-2	C_9_H_7_N_3_S	17.35	189.03	135.01	161.02
Pretilachlor	51218-49-6	C_17_H_26_NO_2_Cl	17.36	162.13	202.12	238.10
Dieldrin	60-57-1	C_12_H_8_CL_6_O	17.44	262.86	81.03	260.86
Oxadiazon	19666-30-9	C_15_H_18_N_2_O_3_Cl_2_	17.51	174.96	302.02	344.07
Iprovalicarb	140923-17-7	C_18_H_28_N_2_O_3_	17.52, 17.82	134.10	116.07	158.12
Carboxin	5234-68-4	C_12_H_13_NO_2_S	17.60	235.07	218.04	143.02
Myclobutanil	88671-89-0	C_15_H_17_ClN_4_	17.60	179.02	150.01	245.06
p,p'-Dichlorodiphenyldichloroethylene	72-55-9	C_14_H_8_Cl_4_	17.64	315.94	247.99	245.99
Buprofezin	69327-76-0	C_16_H_23_N_3_OS	17.68	175.09	171.10	249.11
Imazalil	35554-44-0	C_14_H_14_Cl_2_N_2_O	17.70	174.95	172.96	158.98
Flusilazole	85509-19-9	C_16_H_15_N_3_F_2_Si	17.70	233.06	206.05	314.10
Methoprotryne	841-06-5	C_11_H_21_N_5_OS	17.72	256.12	184.07	212.10
Azaconazole	60207-31-0	C_12_H_11_N_3_O_2_Cl_2_	17.75	216.98	144.96	174.95
Bupirimate	41483-43-6	C_13_H_24_N_4_O_3_S	17.79	208.14	193.14	273.10
Imazamethabenz-methyl	81405-85-8	C_16_H_20_N_2_O_3_	17.82	144.04	176.07	245.09
Kresoxim-methyl	143390-89-0	C_18_H_19_NO_4_	17.83	116.05	131.07	206.08
Metamitron	41394-05-2	C_10_H_10_N_4_O	17.85	174.09	173.08	202.08
Isoxathion	18854-01-8	C_13_H_16_NO_4_PS	17.97	177.02	159.01	313.05
Aramite	140-57-8	C_15_H_23_O_4_SCl	17.98	185.00	175.11	319.08
Nitrofen	1836-75-5	C_12_H_7_Cl_2_NO_3_	18.02	282.98	284.98	202.02
Endrin	72-20-8	C_12_H_8_CL_6_O	18.09	242.95	280.93	316.90
Endrin aldehyde	7421-93-4	C_12_H_8_OCl_6_	18.09	242.95	280.93	344.90
Ancymidol	12771-68-5	C_15_H_16_N_2_O_2_	18.12	228.90	107.02	215.08
Perthan	72-56-0	C_18_H_20_Cl_2_	18.15	223.15	178.08	167.09
Chlorfenapyr	122453-73-0	C_15_H_11_BRCLF_3_N_2_O	18.19	247.05	363.94	361.94
Chloropropylate	5836-10-2	C_17_H_16_O_3_Cl_2_	18.38	138.99	110.99	251.00
Chlorobenzilate	510-15-6	C_16_H_14_O_3_Cl_2_	18.38	138.99	251.00	252.99
Fenthion sulfoxide	3761-41-9	C_10_H_15_O_4_PS_2_	18.51	294.01	278.99	152.98
Diniconazole	83657-24-3	C_15_H_17_N_3_OCl_2_	18.55	268.00	234.04	165.01
Flamprop-isopropyl	52756-22-6	C_19_H_19_NO_3_FCl	18.63	276.06	105.03	156.00
p,p'-Dichlorodiphenyldichloroethane	72-54-8	C_14_H_10_Cl_4_	18.66	235.01	199.03	165.07
Aclonifen	74070-46-5	C_12_H_9_CLN_2_O_3_	18.68	264.03	182.06	212.06
o,p'-Dichlorodiphenyltrichloroethane	789-02-6	C_14_H_9_Cl_5_	18.76	235.00	165.07	237.00
Oxadixyl	77732-09-3	C_14_H_18_N_2_O_4_	18.78	233.09	163.10	132.08
Ethion	563-12-2	C_9_H_22_O_4_P_2_S_4_	18.82	230.97	202.94	153.01
Mepronil	55814-41-0	C_17_H_19_NO_2_	19.07	119.05	210.07	269.14
Triazophos	24017-47-8	C_12_H_16_N_3_O_3_PS	19.22	162.07	257.00	172.09
Azamethiphos	35575-96-3	C_9_H_10_ClN_2_O_5_PS	19.34	182.99	214.97	323.97
Ofurace	58810-48-3	C_14_H_16_NO_3_Cl	19.39	232.10	186.09	281.08
Carbophenothion	786-19-6	C_11_H_16_O_2_PS_3_Cl	19.47	341.97	170.97	199.00
Benalaxyl	71626-11-4	C_20_H_23_NO_3_	19.55	148.11	176.11	206.12
Tepraloxydim	149979-41-9	C_17_H_24_NO_4_Cl	19.55	164.07	136.04	108.04
Diofenolan	63837-33-2	C_18_H_20_O_4_	19.56, 19.77	186.07	131.05	225.09
Cyanofenphos	13067-93-1	C_15_H_14_NO_2_PS	19.60	141.01	169.04	185.02
Edifenphos	17109-49-8	C_14_H_15_O_2_PS_2_	19.60	172.98	186.05	310.02
Quinoxyfen	124495-18-7	C_15_H_8_NOFC_l2_	19.63	306.99	237.06	161.00
Endosulfan sulfate	1031-07-8	C_9_H_6_CL_6_O_4_S	19.69	271.81	236.84	269.81
Propiconazol	60207-90-1	C_15_H_17_Cl_2_N_3_O_2_	19.70, 19.91	172.95	259.03	261.03
Norflurazon	27314-13-2	C_12_H_9_N_3_OF_3_Cl	19.74	303.04	173.03	302.03
Fenhexamid	126833-17-8	C_14_H_17_Cl_2_NO_2_	19.79	176.97	178.97	301.06
p,p'-Dichlorodiphenyltrichloroethane	50-29-3	C_14_H_9_Cl_5_	19.84	235.00	199.03	165.07
Trifloxystrobin	141517-21-7	C_20_H_19_F_3_N_2_O_4_	19.92	116.05	190.05	186.05
Hexazinone	51235-04-2	C_12_H_20_N_4_O_2_	20.17	171.09	71.06	128.08
Tebuconazol	107534-96-3	C_16_H_22_ClN_3_O	20.26	250.07	125.02	163.03
Chloridazon	1698-60-8	C_10_H_8_ClN_3_O	20.26	220.03	221.04	222.02
Nuarimol	63284-71-9	C_17_H_12_N_2_OFCl	20.28	235.03	203.06	314.06
Diclofop-methyl	51338-27-3	C_16_H_14_Cl_2_O_4_	20.39	340.03	254.98	252.98
Triphenyl phosphate	115-86-6	C_18_H_15_O_4_P	20.49	325.06	169.06	233.04
Piperonyl butoxide	51-03-6	C_19_H_30_O_5_	20.62	176.08	161.06	177.09
Oxycarboxin	5259-88-1	C_12_H_13_NO_4_S	20.68	175.01	250.03	267.06
Resmethrin	10453-86-8	C_22_H_26_O_3_	20.70	143.09	128.06	171.08
Zoxamide	156052-68-5	C_14_H_16_NO_2_Cl_3_	20.83	186.97	258.04	242.01
Mefenpyr-diethyl	135590-91-9	C_16_H_18_Cl_2_N_2_O_4_	21.02	271.00	227.01	299.03
Benzoylprop-ethyl	22212-55-1	C_18_H_17_NO_3_Cl_2_	21.12	105.03	292.03	260.02
Spiromesifen	283594-90-1	C_23_H_30_O_4_	21.14	254.13	231.10	226.13
Endrin ketone	53494-70-5	C_12_H_8_OCl_6_	21.16	314.91	281.93	242.95
Fenamiphos sulfone	31972-44-8	C_13_H_22_NO_5_PS	21.27	292.04	320.07	214.06
Bromuconazole	116255-48-2	C13H12BrCl_2_N_3_O	21.31, 22.12	172.95	294.91	174.95
Fenpiclonil	74738-17-3	C_11_H_6_N_2_Cl_2_	21.32	235.99	201.02	237.99
Phosmet	732-11-6	C_11_H_12_NO_4_PS_2_	21.37	160.04	104.03	133.03
Bromopropylate	18181-80-1	C_17_H_16_O_3_Br_2_	21.48	184.94	182.94	338.90
Tetramethrin	7696-12-0	C_19_H_25_NO_4_	21.61	164.07	107.05	123.12
Picolinafen	137641-05-5	C_19_H_12_N_2_O_2_F_4_	21.61	238.05	145.03	376.08
Bifenthrin	82657-04-3	C_23_H_22_ClF_3_O_2_	21.64	181.10	165.07	182.10
Piperophos	24151-93-7	C_14_H_28_NO_3_PS_2_	21.68	122.10	140.11	320.14
4,4′-Methoxychlor	72-43-5	C_16_H_15_O_2_CL_3_	21.73	227.11	212.08	228.11
Bifenazate	149877-41-8	C_17_H_20_N_2_O_3_	21.73	300.15	258.10	196.08
Fenpropathrin	39515-41-8	C_22_H_23_NO_3_	21.83	181.06	209.08	265.07
Etoxazole	153233-91-1	C_21_H_23_F_2_NO_2_	21.91	300.12	187.11	330.13
Tebufenpyrad	119168-77-3	C_18_H_24_N_3_OCl	21.94	333.16	171.03	276.09
Fenamidone	161326-34-7	C_17_H_17_N_3_OS	21.95	268.09	206.07	238.11
Dicofol	115-32-2	C_14_H_9_CL_5_O	21.98	138.99	199.03	140.99
Metconazole	125116-23-6	C_17_H_22_N_3_OCl	21.99	125.02	145.06	250.11
Fenazaquin	120928-09-8	C_20_H_22_N_2_O	22.00	145.10	117.07	160.12
Tetradifon	116-29-0	C_12_H_6_O_2_SCl_4_	22.36	226.89	228.89	158.97
Furathiocarb	65907-30-4	C_18_H_26_N_2_O_5_S	22.54	163.08	194.04	325.13
Phosalone	2310-17-0	C_12_H_15_NO_4_PS_2_Cl	22.68	182.00	121.04	366.99
Pyriproxyfen	95737-68-1	C_20_H_19_NO_3_	22.88	136.08	226.10	137.08
Mirex	2385-85-5	C_10_Cl_12_	22.96	271.81	269.81	331.81
Mefenacet	73250-68-7	C_16_H_14_N_2_O_2_S	23.04	192.01	136.02	120.08
Cyhalothrin	68085-85-8	C_23_H_19_ClF_3_NO_3_	23.13, 23.49	141.05	197.03	161.06
Tralkoxydim	87820-88-0	C_20_H_27_NO_3_	23.17	137.04	227.13	283.16
Fenarimol	60168-88-9	C_17_H_12_N_2_OCl_2_	23.58	251.00	219.03	252.99
Trifenmorph	1420-06-0	C_23_H_23_NO	23.65	243.12	228.09	239.09
Azinphos-ethyl	2642-71-9	C_12_H_16_N_3_O_3_PS_2_	23.85	132.04	104.05	160.05
Pyrazophos	13457-18-6	C_14_H_20_N_3_O_5_PS	23.88	221.08	265.09	193.05
Acrinathrin	101007-06-1	C_26_H_21_F_6_NO_5_	23.90	181.06	208.08	289.07
Fluoroglycofen-ethyl	77501-90-7	C_18_H_13_NO_7_F_3_Cl	23.96	343.99	223.04	447.03
Fenoxaprop-ethyl	66441-23-4	C_18_H_16_NO_5_Cl	24.25	288.04	182.06	361.07
Bitertanol	55179-31-2	C_20_H_23_N_3_O_2_	24.59	170.07	168.11	171.08
Spirodiclofen	148477-71-8	C_21_H_24_Cl_2_O_4_	24.74	259.05	312.03	156.96
Permethrin	61949-76-6	C_21_H_20_CL_2_O_3_	24.78, 25.03	183.08	163.01	127.03
Pyridaben	96489-71-3	C_19_H_25_ClN_2_OS	24.96	147.12	117.07	309.08
Fluquinconazole	136426-54-5	C_16_H_8_N_5_OFC_l2_	25.13	340.04	298.02	286.02
Coumaphos	56-72-4	C_14_H_16_CLO_5_PS	25.16	362.01	210.01	225.98
Prochloraz	67747-09-5	C_15_H_16_N_3_O_2_Cl_3_	25.28	180.11	265.95	308.00
Butafenacil	134605-64-4	C_20_H_18_N_2_O_6_F_3_Cl	25.63	331.01	123.99	179.98
Prallethrin	23031-36-9	C_19_H_24_O_3_	25.83	123.12	81.07	105.07
Cyfluthrin	68359-37-5	C_22_H_18_NO_3_FCl_2_	25.94, 26.13, 26.26, 26.35	206.06	199.06	163.01
Cypermethrin	52315-07-8	C_22_H_19_Cl_2_NO_3_	26.51, 26.71, 26.83, 26.93	181.06	163.01	127.03
Boscalid	188425-85-6	C_18_H_12_Cl_2_N_2_O	26.53	342.03	111.99	139.99
Quizalofop-ethyl	76578-14-8	C_19_H_17_CLN_2_O_4_	26.75	372.09	243.03	163.01
Flucythrinate	70124-77-5	C_26_H_23_F_2_NO_4_	26.93, 27.30	157.05	199.09	225.08
Etofenprox	80844-07-1	C_25_H_28_O_3_	27.03	163.11	135.08	164.12
Pyridalyl	179101-81-6	C_18_H_14_NO_3_F_3_Cl_4_	27.18	204.06	148.04	176.03
Fenvalerate	51630-58-1	C_25_H_22_NO_3_Cl	28.21, 28.61	419.13	125.02	167.06
Flumioxazin	103361-09-7	C_19_H_15_FN_2_O_4_	28.25	354.10	259.05	326.11
Pyraclostrobin	175013-18-0	C_19_H_18_N_3_O_4_Cl	28.34	132.04	104.05	164.07
Tau-fluvalinate	102851-06-9	C_26_H_22_ClF_3_N_2_O_3_	28.62, 28.75	250.06	252.06	205.99
Difenoconazole	119446-68-3	C_19_H_17_N_3_O_3_Cl_2_	28.95, 29.07	323.02	266.98	264.98
Deltamethrin	52918-63-5	C_22_H_19_Br_2_NO_3_	29.21, 29.56	171.99	173.99	252.90
Azoxystrobin	131860-33-8	C_22_H_17_N_3_O_5_	30.02	344.10	372.10	388.09
Dimethomorph	110488-70-5	C_21_H_22_ClNO_4_	30.06	301.06	303.06	387.12

**Table 2:** Chemical substances with high detection rate detected in chrysanthemum samples.

Number	Pesticides	Number of detected samples	Detection rate	Value range (*μ*g/kg)
1	Profenofos	50	31.5	0.12–40.8
2	Procymidone	18	14.5	0.10–177.9
3	Metalaxyl	36	24.5	0.29–108.9
4	Chlorfenapyr	48	26.0	0.25–35.7
5	Difenoconazole	39	19.5	0.27–40.5
6	Dimethomorph	33	16.5	0.18–280.4
7	Cypermethrin	42	21.0	6.2–199.6
8	Tebuconazol	29	14.5	0.45–76.8
9	Propiconazol	36	18.5	0.15–49.5
10	Pyrimethanil	44	22.0	1.2–110.4

## Data Availability

The data used to support the findings of this study are available from the corresponding author upon request.

## Supplementary Materials

Supplementary Materials Table S1 is included in the supplementary file.

## References

[1] Lin L.-Z., Harnly J. M. (2010). Identification of the phenolic components of chrysanthemum flower (chrysanthemum morifolium ramat). *Food Chemistry*.

[2] Chu Q., Fu L., Guan Y., Ye J. (2004). Determination and differentiation of flos Chrysanthemum based on characteristic electrochemical profiles by capillary electrophoresis with electrochemical detection. *Journal of Agricultural and Food Chemistry*.

[3] Wu Y., Wang X., Xue J., Fan E. (2017). Plant phenolics extraction from flos chrysanthemi: response surface methodology based optimization and the correlation between extracts and free radical Scavenging activity. *Journal of Food Science*.

[4] Wu J., Wei H., Xue J. (2012). Degradation of imidacloprid in chrysanthemi flos and soil. *Bulletin of Environmental Contamination and Toxicology*.

[5] Shamsipur M., Yazdanfar N., Ghambarian M. (2016). Combination of solid-phase extraction with dispersive liquid–liquid microextraction followed by GC–MS for determination of pesticide residues from water, milk, honey and fruit juice. *Food Chemistry*.

[6] Abdulra’uf L. B., Tan G. H. (2015). Chemometric approach to the optimization of HS-SPME/GC-MS for the determination of multiclass pesticide residues in fruits and vegetables. *Food Chemistry*.

[7] Zhu B., Xu X., Luo J. (2019). Simultaneous determination of 131 pesticides in tea by on-line GPC-GC-MS/MS using graphitized multi-walled carbon nanotubes as dispersive solid phase extraction sorbent. *Food Chemistry*.

[8] Golge O., Kabak B. (2015). Evaluation of QuEChERS sample preparation and liquid chromatography-triple-quadrupole mass spectrometry method for the determination of 109 pesticide residues in tomatoes. *Food Chemistry*.

[9] He J., Zhang B., Zhang H. (2019). Monitoring of 49 pesticides and 17 mycotoxins in wine by QuEChERS and UHPLC-MS/MS analysis. *Journal of Food Science*.

[10] Huang Y., Shi T., Luo X. (2019). Determination of multi-pesticide residues in green tea with a modified QuEChERS protocol coupled to HPLC-MS/MS. *Food Chemistry*.

[11] Garcia C. V., Gotah A. (2017). Application of QuEChERS for determining xenobiotics in foods of animal origin. *Journal of Analytical Methods in Chemistry*.

[12] Mekonnen B., Siraj J., Negash S. (2021). Determination of pesticide residues in food premises using QuECHERS method in Bench-Sheko Zone, Southwest Ethiopia. *BioMed Research International*.

[13] González-Curbelo M., Herrera-Herrera A. V., Hernández-Borges J., Rodríguez-Delgado M. (2013). Analysis of pesticides residues in environmental water samples using multiwalled carbon nanotubes dispersive solid-phase extraction. *Journal of Separation Science*.

[14] Han Y., Zou N., Song L. (2015). Simultaneous determination of 70 pesticide residues in leek, leaf lettuce and garland chrysanthemum using modified QuEChERS method with multi-walled carbon nanotubes as reversed-dispersive solid-phase extraction materials. *Journal of Chromatography B*.

[15] Zhao P., Wang L., Zhou L., Zhang F., Kang S., Pan C. (2012). Multi-walled carbon nanotubes as alternative reversed-dispersive solid phase extraction materials in pesticide multi-residue analysis with QuEChERS method. *Journal of Chromatography A*.

[16] Lu X.-Y., Ouyang Y.-Q., Zeng W.-Y. (2021). Effect of pretreatment on detection of 37 pesticide residues in chrysanthemum indicum. *Journal of Analytical Methods in Chemistry*.

[17] Tan P., Xu L., Wei X.-C. (2021). Rapid screening and quantitative analysis of 74 pesticide residues in Herb by retention index combined with GC-QQQ-MS/MS. *Journal of Analytical Methods in Chemistry*.

[18] Zhou G.-W., Li Y.-M., Liu C.-N., Ren H.-M., Li H.-Y. (2021). Rapid simultaneous determination of 43 pesticide residues in schizonepeta tenuifolia by gas chromatography mass spectrometry. *International Journal of Analytical Chemistry*.

[19] Chen J.-N., Lian Y.-J., Zhou Y.-R. (2019). Determination of 107 pesticide residues in Wolfberry with acetate-buffered salt extraction and Sin-QuEChERS nano column purification coupled with ultra performance liquid chromatography tandem mass spectrometry. *Molecules*.

[20] Li Y., An Q., Zhang C., Pan C., Zhang Z. (2020). Comparison of Sin-QuEChERS nano and d-SPE methods for pesticide multi-residues in lettuce and Chinese chives. *Molecules*.

[21] Meng Z., Li Q., Cong J. (2021). Rapid screening of 350 pesticide residues in vegetable and fruit juices by multi-plug filtration cleanup method combined with gas chromatography-electrostatic field orbitrap high resolution mass spectrometry. *Foods*.

[22] Tienstra M., Mol H. G. J. (2018). Application of gas chromatography coupled to quadrupole-orbitrap mass spectrometry for pesticide residue analysis in cereals and feed ingredients. *Journal of AOAC International*.

[23] Moreno-González D., Alcántara-Durán J., Addona S. M., Beneito-Cambra M. (2018). Multi-residue pesticide analysis in virgin olive oil by nanoflow liquid chromatography high resolution mass spectrometry. *Journal of Chromatography A*.

[24] Saito-Shida S., Hamasaka T., Nemoto S., Akiyama H. (2018). Multiresidue determination of pesticides in tea by liquid chromatography-high-resolution mass spectrometry: comparison between orbitrap and time-of-flight mass analyzers. *Food Chemistry*.

[25] Wang Z., Cao Y., Ge N. (2016). Wide-scope screening of pesticides in fruits and vegetables using information-dependent acquisition employing UHPLC-QTOF-MS and automated MS/MS library searching. *Analytical and Bioanalytical Chemistry*.

[26] Cheng Z., Zhang X., Geng X. (2018). A target screening method for detection of organic pollutants in fruits and vegetables by atmospheric pressure gas chromatography quadrupole-time-of-flight mass spectrometry combined with informatics platform. *Journal of Chromatography A*.

