# Supplementary material for: Rapid Screening of 352 Pesticide Residues in Chrysanthemum Flower by Gas Chromatography Coupled to Quadrupole-Orbitrap Mass Spectrometry with Sin-QuEChERS Nanocolumn Extraction
Source: J Anal Methods Chem. 2022 Jun 15;2022:7684432. doi: 10.1155/2022/7684432 (PMC9217587; doi:10.1155/2022/7684432)
Supplement: Supplementary Materials — Table S1 is included in the supplementary file. [file 7684432.f1.docx]

**Supporting Information**

**For**

**Rapid Screening of 352 Pesticide Residues in Chrysanthemum Flower by Gas Chromatography Coupled to Quadrupole Orbitrap Mass Spectrometry with Sin-QuEChERS Nano Column Extraction**

Zhijuan Meng^b^, Chunyan Su^c^, Sufang Fan^b^, Yan Li^b^, Haiye Liu^a^, Xuan Zhang^a^, Pingping Chen^a^, Yunyun Geng^a^, Qiang Li^b,*^, Yuanyuan Wang^a,*^

^a^ Department of Pharmacology, College of Basic Medicine, Hebei University of Chinese Medicine, Shijiazhuang, 050200, Hebei Province, China.

^b^ Hebei Food Inspection and Research Institute, Key Laboratory of Food Safety of Hebei Province, Shijiazhuang, 050091, Hebei Province, China

^c^ School of Pharmacy, Hebei University of Chinese Medicine, Shijiazhuang, 050200, Hebei Province, China.

* Corresponding author. Emails: liqiang@nepp.com.cn; wangyy0830@iccas.ac.cn

**Table S1.** Matrix effects (ME), Correlation coefficients (r), LODs, LOQs and spiked recoveries (RSD) of the 352 pesticides in chrysanthemum flower (n=6).

| **Pesticides** |  |  |  |  |  |  | **Recoveries, % (RSD, %)** | | |
| --- | --- | --- | --- | --- | --- | --- | --- | --- | --- |
|  | **ME^a^** | **ME^b^** | **ME^c^** | **Correlation coefficient** | **LOD** | **LOQ** | **10** | **50** | **100** |
|  |  |  |  | ***r*** | **(μg·kg^-1^)** | **(μg·kg^-1^)** | **μg·kg^-1^** | **μg·kg^-1^** | **μg·kg^-1^** |
| Clopyralid | 1.5 | 1.51 | 1.61 | 0.9925 | 3 | 10 | 78.4(9.1) | 78.0(6.2) | 82.1(7.1) |
| Dichlorvos | 1.23 | 1.24 | 1.25 | 0.999 | 3 | 10 | 73.4(7.1) | 74.2(9.3) | 79.1(9.5) |
| Methamidophos | 1.35 | 1.42 | 1.5 | 0.999 | 1 | 3 | 73.2(9.2) | 78.3(8.2) | 79.7(4.3) |
| Thiofanox | 1.11 | 1.16 | 1.95 | 0.9989 | 1 | 3 | 78.2(7.2) | 83.1(9.3) | 88.4(6.1) |
| Allidochlor | 1.13 | 1.15 | 1.76 | 0.9957 | 0.3 | 1 | 91.2(6.4) | 92.1(4.2) | 95.5(4.0) |
| Dichlobenil | 1.02 | 1.41 | 1.73 | 0.9907 | 0.3 | 1 | 103.8(7.1) | 104.1(4.0) | 97.2(6.3) |
| EPTC | 1.21 | 1.33 | 1.61 | 0.9928 | 0.3 | 1 | 95.4(6.1) | 95.3(4.3) | 88.5(7.4) |
| Dichlormid | 1.07 | 1.29 | 1.33 | 0.9935 | 0.3 | 1 | 102.4(6.2) | 87.4(5.3) | 103.4(6.2) |
| 2,4,6-Trichlorophenol | 1.21 | 1.38 | 1.87 | 0.9944 | 0.5 | 2 | 94.3(9.2) | 84.3(5.4) | 94.3(6.2) |
| 3,5-Dichloroaniline | 1.06 | 1.08 | 1.23 | 0.9985 | 0.5 | 2 | 107.2(8.1) | 106.2(6.5) | 88.4(9.5) |
| O-Phthalimide | 1.23 | 1.29 | 1.86 | 0.9943 | 0.3 | 1 | 104.3(5.1) | 89.4(4.2) | 93.1(8.2) |
| Mevinphos | 1.6 | 1.59 | 1.62 | 0.9979 | 1 | 3 | 74.2(6.4) | 87.2(7.4) | 103.3(8.1) |
| Acephate | 1.04 | 1.41 | 1.36 | 0.9902 | 0.3 | 1 | 101.4(6.1) | 109.4(9.1) | 86.3(5.5) |
| Vernolate | 1.42 | 1.32 | 1.34 | 0.9995 | 0.3 | 1 | 105.3(8.1) | 76.5(8.1) | 93.2(9.2) |
| Propham | 1.24 | 1.42 | 1.91 | 0.9931 | 0.3 | 1 | 94.5(7.2) | 110.2(8.1) | 97.2(5.2) |
| Etridiazole | 1.11 | 1.28 | 1.35 | 0.9911 | 1 | 3 | 107.1(7.5) | 87.3(7.4) | 105.1(7.1) |
| Pebulate | 1.32 | 1.56 | 1.84 | 0.9937 | 1 | 3 | 101.3(5.2) | 79.2(9.1) | 102.1(5.2) |
| cis-1,2,3,6-Tetrahydrophthalimide | 1.23 | 1.65 | 1.09 | 0.9958 | 0.5 | 2 | 96.5(5.5) | 83.4(9.2) | 93.4(9.3) |
| Chloroneb | 1.23 | 1.48 | 1.87 | 0.9993 | 1 | 3 | 104.3(7.4) | 80.5(9.6) | 109.2(7.3) |
| Tebuthiuron | 1.12 | 1.75 | 1.41 | 0.9979 | 0.3 | 1 | 107.4(7.4) | 85.5(9.3) | 104.1(7.2) |
| Fenobucarb | 1.03 | 1.21 | 1.55 | 0.9989 | 1 | 3 | 95.1(6.3) | 93.2(9.1) | 94.5(6.4) |
| Pentachlorobenzene | 1.1 | 1.18 | 1.64 | 0.9968 | 0.5 | 2 | 89.1(8.2) | 74.3(9.4) | 102.5(9.6) |
| Isoprocarb | 1.57 | 1.63 | 1.59 | 0.9952 | 0.5 | 2 | 92.4(6.5) | 75.6(9.7) | 84.8(6.9) |
| Molinate | 1.44 | 1.44 | 1.16 | 0.9952 | 0.5 | 2 | 85.0(7.1) | 107.2(9.3) | 123.4(9.5) |
| Heptenophos | 1.4 | 1.33 | 1.78 | 0.992 | 0.5 | 2 | 95.4(6.1) | 80.2(9.4) | 105.1(7.4) |
| Chlorfenprop-methyl | 1.19 | 1.28 | 1.26 | 0.9955 | 1 | 3 | 94.3(9.4) | 73.5(9.6) | 84.7(8.2) |
| Omethoate | 1.33 | 1.72 | 1.75 | 0.9993 | 1 | 3 | 84.6(7.3) | 75.8(9.2) | 94.0(8.1) |
| Propoxur | 1.13 | 1.34 | 1.55 | 0.9951 | 0.3 | 1 | 90.5(8.4) | 99.5(9.1) | 105.2(4.3) |
| Tecnazene | 1.24 | 1.34 | 1.37 | 0.9946 | 0.5 | 2 | 105.3(7.1) | 81.2(9.5) | 106.5(7.4) |
| Propachlor | 1.5 | 1.11 | 1.12 | 0.9951 | 1 | 3 | 92.5(6.6) | 90.4(4.2) | 94.5(5.3) |
| Diphenylamine | 1.03 | 1.11 | 1.93 | 0.9962 | 0.3 | 1 | 93.4(6.1) | 94.2(4.5) | 96.9(6.2) |
| Ethoprophos | 1.39 | 1.72 | 1.7 | 0.9952 | 3 | 10 | 103.4(4.5) | 75.6(9.7) | 84.2(8.3) |
| Tributyl phosphate | 1.86 | 1.63 | 1.63 | 0.996 | 0.3 | 1 | 104.2(7.3) | 87.4(8.2) | 101.4(5.3) |
| Cycloate | 1.47 | 1.58 | 1.47 | 0.9973 | 0.5 | 2 | 109.5(7.6) | 92.7(4.8) | 93.3(8.2) |
| 2,3,5,6-tetrachloroaniline | 1.21 | 1.08 | 1.32 | 0.9929 | 1 | 3 | 97.4(9.5) | 84.6(9.7) | 108.8(9.3) |
| Atrazine-desethyl | 1.18 | 1.36 | 2.07 | 0.9949 | 0.3 | 1 | 101.1(4.3) | 94.4(6.3) | 95.5(4.2) |
| Dicrotophos | 1.36 | 1.58 | 1.41 | 0.995 | 1 | 3 | 85.3(4.4) | 84.5(8.1) | 107.3(5.2) |
| Methabenzthiazuron | 1.13 | 1.57 | 1.21 | 0.9916 | 0.5 | 2 | 94.2(8.2) | 97.1(4.4) | 108.6(8.3) |
| Triﬂuralin | 1.03 | 1.62 | 1.16 | 0.9926 | 0.3 | 1 | 104.5(4.4) | 83.5(5.3) | 109.4(9.5) |
| Bendiocarb | 1.44 | 1.3 | 1.42 | 0.9999 | 0.5 | 2 | 92.3(8.2) | 102.4(9.4) | 95.3(6.4) |
| Benﬂuralin | 1.36 | 1.39 | 1.48 | 0.9974 | 0.3 | 1 | 107.5(9.4) | 96.5(7.1) | 88.3(4.2) |
| Sulfotep | 1.03 | 1.07 | 1.65 | 0.997 | 0.5 | 2 | 92.3(7.4) | 98.4(7.2) | 95.4(6.3) |
| Cadusafos | 1.1 | 1.17 | 1.32 | 0.9927 | 0.3 | 1 | 103.5(9.5) | 78.4(9.1) | 92.2(7.3) |
| Tebutam | 1.02 | 1.05 | 2 | 0.9949 | 3 | 10 | 110.3(8.1) | 97.2(9.4) | 96.3(7.3) |
| Promecarb | 1.1 | 1.73 | 2.1 | 0.9991 | 1 | 3 | 80.2(9.2) | 72.8(9.2) | 99.3(8.3) |
| Phorate | 1.36 | 1.43 | 1.58 | 0.9914 | 0.5 | 2 | 95.2(6.3) | 90.4(9.5) | 77.6(11.3) |
| α-Hexachlorocyclohexane | 1.35 | 1.34 | 1.43 | 0.9974 | 1 | 3 | 96.5(5.4) | 80.3(4.3) | 109.5(9.6) |
| Atratone | 1.07 | 1.45 | 1.4 | 0.995 | 3 | 10 | 95.5(8.6) | 92.3(8.5) | 95.5(9.0) |
| 3,4,5-Trimethacarb | 1.06 | 1.14 | 1.21 | 0.9925 | 1 | 3 | 81.4(4.4) | 93.5(8.6) | 86.7(6.8) |
| Dicloran | 1.14 | 1.23 | 1.57 | 0.9935 | 3 | 10 | 95.1(6.3) | 97.2(7.4) | 96.5(8.6) |
| Pentachloroanisole | 1.46 | 1.51 | 1.93 | 0.9992 | 3 | 10 | 78.8(9.6) | 78.4(6.3) | 82.8(7.2) |
| Ethoxyquin | 1.52 | 1.71 | 1.66 | 0.9958 | 3 | 10 | 75.5(9.2) | 73.4(8.9) | 78.1(8.2) |
| Prometon | 1.14 | 1.58 | 1.96 | 0.9991 | 1 | 3 | 82.8(5.1) | 89.3(2.2) | 89.2(5.3) |
| Atrazine | 1.12 | 1.35 | 1.61 | 0.9945 | 1 | 3 | 88.2(8.4) | 82.3(9.3) | 93.2(6.1) |
| Monolinuron | 1.4 | 1.17 | 1.45 | 0.9905 | 0.3 | 1 | 92.5(6.6) | 93.5(5.4) | 95.3(4.5) |
| Propazine | 1.14 | 1.29 | 1.56 | 0.991 | 0.3 | 1 | 105.5(7.3) | 105.2(4.4) | 98.5(7.2) |
| Clomazone | 1.11 | 1.35 | 1.76 | 0.9935 | 0.3 | 1 | 95.3(6.1) | 95.6(4.4) | 88.5(7.2) |
| Terbumeton | 1.29 | 1.49 | 1.83 | 0.997 | 0.3 | 1 | 102.3(7.4) | 87.5(7.4) | 103.1(8.3) |
| β-Hexachlorocyclohexane | 1.55 | 1.57 | 1.63 | 0.9987 | 0.5 | 2 | 93.2(9.2) | 84.1(6.2) | 93.2(7.3) |
| Aminocarb | 1.22 | 1.41 | 1.34 | 0.992 | 0.5 | 2 | 106.1(8.3) | 106.1(6.4) | 88.1(9.2) |
| Isocarbamid | 1.25 | 1.22 | 1.8 | 0.9993 | 0.3 | 1 | 104.5(7.1) | 89.4(5.2) | 93.5(8.3) |
| Cyromazine | 1.52 | 1.73 | 1.93 | 0.9931 | 1 | 3 | 78.2(6.3) | 87.5(7.2) | 103.5(8.4) |
| γ-Hexachlorocyclohexane | 1.47 | 1.73 | 1.9 | 0.9934 | 0.3 | 1 | 101.3(7.2) | 108.3(9.2) | 86.9(5.3) |
| Propetamphos | 1.43 | 1.46 | 1.75 | 0.9984 | 0.3 | 1 | 105.3(8.2) | 76.8(9.1) | 93.4(8.2) |
| Cycluron | 1.11 | 1.62 | 1.66 | 0.9933 | 0.3 | 1 | 94.5(7.1) | 112.6(8.2) | 97.7(5.3) |
| Terbuthylazine | 1.51 | 1.64 | 2.07 | 0.9919 | 1 | 3 | 108.5(7.1) | 87.4(7.3) | 105.3(8.1) |
| Terbufos | 1.13 | 1.08 | 1.16 | 0.9915 | 1 | 3 | 101.2(6.2) | 79.1(9.1) | 102.2(7.2) |
| Cyanophos | 1.02 | 1.1 | 1.13 | 0.999 | 0.5 | 2 | 96.2(5.2) | 83.3(9.1) | 93.1(7.2) |
| Trietazine | 1.49 | 1.66 | 1.99 | 0.997 | 1 | 3 | 104.1(7.3) | 80.2(8.7) | 109.3(7.2) |
| Quintozene | 1.51 | 1.55 | 1.98 | 0.9957 | 3 | 10 | 75.4(9.1) | 78.0(6.2) | 82.1(7.1) |
| Fonofos | 1.52 | 1.72 | 1.99 | 0.9916 | 3 | 10 | 73.8(6.1) | 74.2(9.3) | 79.1(9.5) |
| Pyroquilon | 1.43 | 1.79 | 1.91 | 0.9983 | 1 | 3 | 74.2(8.2) | 78.3(8.2) | 79.7(4.3) |
| Dinoterb | 1.06 | 1.54 | 1.41 | 0.9914 | 1 | 3 | 78.2(7.2) | 83.1(9.3) | 88.4(6.1) |
| Pyrimethanil | 1.09 | 1.49 | 1.46 | 0.9996 | 0.3 | 1 | 94.2(6.9) | 92.1(4.2) | 95.5(4.0) |
| Diazinon | 1.25 | 2.17 | 1.84 | 0.9959 | 0.3 | 1 | 103.5(6.1) | 104.1(4.0) | 97.2(6.3) |
| Flufenoxuron | 1.19 | 1.28 | 1.46 | 0.9945 | 0.3 | 1 | 95.4(6.1) | 95.3(4.3) | 88.5(7.4) |
| Disulfoton | 1.16 | 1.66 | 1.09 | 0.9903 | 0.3 | 1 | 102.3(3.2) | 87.4(5.3) | 103.4(6.2) |
| Paraoxon-methyl | 1.05 | 1.78 | 1.69 | 0.9905 | 0.5 | 2 | 94.8(9.2) | 84.3(5.4) | 94.3(6.2) |
| Secbumeton | 1.28 | 1.78 | 1.17 | 0.9948 | 0.5 | 2 | 105.2(8.1) | 106.2(6.5) | 88.4(9.5) |
| Aziprotryne | 1.55 | 1.48 | 1.47 | 0.9906 | 0.3 | 1 | 104.3(5.1) | 89.4(4.2) | 93.1(8.2) |
| Dinitramine | 1.32 | 1.11 | 1.39 | 0.9989 | 1 | 3 | 74.2(6.4) | 87.2(7.4) | 103.3(8.1) |
| Fenfuram | 1.37 | 1.15 | 1.6 | 0.999 | 0.3 | 1 | 101.4(6.1) | 109.4(9.1) | 86.3(5.5) |
| δ-Hexachlorocyclohexane | 1.47 | 1.44 | 1.98 | 0.9963 | 0.3 | 1 | 105.3(5.1) | 76.5(8.1) | 93.2(9.2) |
| Mexacarbate | 1.34 | 1.74 | 1.71 | 0.9936 | 0.3 | 1 | 94.5(7.2) | 110.2(8.1) | 97.2(5.2) |
| Isazofos | 1.22 | 1.05 | 1.26 | 0.9957 | 1 | 3 | 108.1(7.5) | 87.3(7.4) | 105.1(7.1) |
| Chlorothalonil | 1.26 | 1.76 | 2.11 | 0.9983 | 1 | 3 | 103.3(5.2) | 79.2(9.1) | 102.1(5.2) |
| Triallate | 1.29 | 1.27 | 1.7 | 0.9988 | 0.5 | 2 | 96.5(5.5) | 83.4(9.2) | 93.4(9.3) |
| Tebupirimfos | 1.42 | 1.22 | 1.42 | 0.9983 | 0.3 | 1 | 105.3(7.4) | 80.5(9.6) | 109.2(7.3) |
| musk ambrette | 1.03 | 1.49 | 1.58 | 0.9955 | 0.3 | 1 | 106.4(7.4) | 85.5(9.3) | 104.1(7.2) |
| Oxabetrinil | 1.24 | 1.49 | 1.53 | 0.9954 | 1 | 3 | 95.1(4.3) | 93.2(9.1) | 94.5(6.4) |
| Iprobenfos | 1.44 | 1.17 | 1.88 | 0.9914 | 0.5 | 2 | 89.1(8.2) | 74.3(9.4) | 102.5(9.6) |
| Fluroxypyr | 1.56 | 1.69 | 1.48 | 0.9968 | 0.5 | 2 | 92.4(6.5) | 75.6(9.7) | 84.8(6.9) |
| Pirimicarb | 1.51 | 1.47 | 1.52 | 0.9978 | 0.5 | 2 | 85.0(7.1) | 107.2(9.3) | 123.4(9.5) |
| Monalide | 1.41 | 1.74 | 1.87 | 0.9992 | 0.5 | 2 | 95.4(6.1) | 80.2(9.4) | 105.1(7.4) |
| Furmecyclox | 1.56 | 1.73 | 1.97 | 0.9981 | 1 | 3 | 94.3(7.4) | 73.5(9.6) | 84.7(8.2) |
| Benoxacor | 1.16 | 1.16 | 1.73 | 0.9947 | 1 | 3 | 84.6(8.3) | 75.8(9.2) | 94.0(8.1) |
| Pentachloroaniline | 1.31 | 1.35 | 1.9 | 0.9913 | 0.3 | 1 | 92.5(8.4) | 99.5(9.1) | 105.2(4.3) |
| Benfuresate | 1.33 | 1.57 | 1.16 | 0.995 | 0.5 | 2 | 105.3(7.1) | 81.2(9.5) | 106.5(7.4) |
| Dioxacarb | 1.42 | 1.65 | 1.54 | 0.9945 | 1 | 3 | 92.5(6.6) | 90.4(4.2) | 94.5(5.3) |
| Cyprazine | 1.04 | 1.31 | 1.69 | 0.9997 | 0.3 | 1 | 93.4(6.1) | 94.2(4.5) | 96.9(6.2) |
| Phosphamidon | 1.29 | 1.36 | 1.77 | 0.9931 | 3 | 10 | 103.4(4.5) | 75.6(9.7) | 84.2(8.3) |
| Dichlorprop | 1.31 | 1.25 | 1.77 | 0.9917 | 0.3 | 1 | 104.2(7.3) | 87.4(8.2) | 101.4(5.3) |
| Dichlofenthion | 1.45 | 1.47 | 1.77 | 0.9941 | 0.5 | 2 | 109.5(7.6) | 92.7(4.8) | 93.3(8.2) |
| Fenthion | 1.6 | 1.59 | 1.33 | 0.997 | 1 | 3 | 97.5(9.5) | 84.6(9.7) | 108.8(9.3) |
| Propanil | 1.19 | 1.49 | 1.15 | 0.997 | 0.3 | 1 | 101.1(4.3) | 94.4(6.3) | 95.5(4.2) |
| 2,4-DB | 1.58 | 1.63 | 1.73 | 0.9901 | 1 | 3 | 87.3(4.4) | 84.5(8.1) | 107.3(5.2) |
| Chlorthiamid | 1.12 | 1.14 | 1.11 | 0.9909 | 0.5 | 2 | 94.2(8.2) | 97.1(4.4) | 108.6(8.3) |
| Dimethachlor | 1.36 | 1.25 | 1.57 | 0.9932 | 0.3 | 1 | 106.5(4.4) | 83.5(5.3) | 109.4(9.5) |
| Metribuzin | 1.37 | 1.78 | 1.65 | 0.9991 | 0.5 | 2 | 92.3(5.2) | 102.4(9.4) | 95.3(6.4) |
| Dimethenamid | 1.34 | 1.5 | 1.45 | 0.9957 | 0.3 | 1 | 107.5(8.4) | 96.5(7.1) | 88.3(4.2) |
| Bromobutide | 1.51 | 1.63 | 1.74 | 0.9965 | 0.5 | 2 | 96.3(7.4) | 98.4(7.2) | 95.4(6.3) |
| Terbucarb | 1.42 | 1.6 | 1.7 | 0.9912 | 0.3 | 1 | 102.5(9.5) | 78.4(9.1) | 92.2(7.3) |
| Malaoxon | 1.45 | 1.21 | 1.3 | 0.9925 | 0.5 | 2 | 110.3(8.1) | 97.2(9.4) | 96.3(7.3) |
| Vinclozolin | 1.06 | 1.23 | 1.76 | 0.9923 | 1 | 3 | 86.2(9.2) | 72.8(9.2) | 99.3(8.3) |
| Parathion-methyl | 1.44 | 1.54 | 1.49 | 0.9995 | 0.5 | 2 | 95.1(6.3) | 90.4(9.5) | 77.6(11.3) |
| Chlorpyrifos-methyl | 1.46 | 1.55 | 1.66 | 0.9953 | 1 | 3 | 96.5(5.4) | 80.3(4.3) | 109.5(9.6) |
| Transfluthrin | 1.32 | 1.38 | 1.36 | 0.9952 | 3 | 10 | 95.5(7.6) | 92.3(8.5) | 95.5(9.0) |
| Simetryn | 1.21 | 1.38 | 1.48 | 0.9901 | 1 | 3 | 81.4(8.4) | 93.5(8.6) | 86.7(6.8) |
| Fuberidazole | 1.04 | 1.15 | 1.16 | 0.9941 | 3 | 10 | 95.1(6.3) | 97.2(7.4) | 96.5(8.6) |
| Tolclofos-methyl | 1.19 | 1.78 | 1.33 | 0.9948 | 1 | 3 | 78.6(9.6) | 78.4(6.3) | 82.8(7.2) |
| Alachlor | 1.49 | 1.6 | 1.54 | 0.998 | 3 | 10 | 75.5(9.2) | 73.4(8.9) | 78.1(8.2) |
| Ametryn | 1.39 | 1.47 | 1.32 | 0.9939 | 1 | 3 | 82.7(5.1) | 89.3(6.2) | 89.2(5.3) |
| Heptachlor | 1.09 | 1.16 | 1.14 | 0.9953 | 1 | 3 | 88.2(8.4) | 82.3(9.3) | 93.2(6.1) |
| Prometryn | 1.08 | 1.3 | 1.71 | 0.9922 | 0.3 | 1 | 92.5(5.6) | 93.5(5.4) | 95.3(4.5) |
| Acetochlor | 1.19 | 1.06 | 1.22 | 0.995 | 0.3 | 1 | 105.5(7.3) | 105.2(4.4) | 98.5(7.2) |
| Paraoxon-ethyl | 1.13 | 1.57 | 1.52 | 0.9997 | 0.3 | 1 | 95.3(6.1) | 95.6(4.4) | 88.5(7.2) |
| Metalaxyl | 1.24 | 1.53 | 1.24 | 0.9908 | 0.3 | 1 | 102.3(8.4) | 87.5(7.4) | 103.1(8.3) |
| Tridiphane | 1.53 | 1.14 | 1.22 | 0.9906 | 0.5 | 2 | 93.9(9.2) | 84.1(6.2) | 93.2(7.3) |
| Octachlorodipropyl ether | 1.51 | 1.77 | 1.34 | 0.9984 | 0.5 | 2 | 106.7(8.3) | 106.1(6.4) | 88.1(9.2) |
| Prosulfocarb | 1.4 | 1.73 | 1.42 | 0.9973 | 0.3 | 1 | 104.5(7.1) | 89.4(5.2) | 93.5(8.3) |
| Fenpropidin | 1.32 | 1.53 | 1.6 | 0.9906 | 1 | 3 | 78.2(6.3) | 87.5(7.2) | 103.5(8.4) |
| 1-Naphthylacetamide | 1.03 | 1.67 | 1.57 | 0.9988 | 0.3 | 1 | 101.3(6.2) | 108.3(9.2) | 86.9(5.3) |
| Dithiopyr | 1.3 | 1.57 | 1.58 | 0.9984 | 0.3 | 1 | 105.3(8.2) | 76.8(9.1) | 93.4(8.2) |
| Orbencarb | 1.56 | 1.27 | 1.24 | 0.9988 | 0.3 | 1 | 94.5(9.1) | 112.6(8.2) | 97.7(5.3) |
| Terbutryn | 1.21 | 1.5 | 1.53 | 0.9932 | 1 | 3 | 108.5(7.1) | 87.4(7.3) | 105.3(8.1) |
| Spiroxamine | 1.34 | 1.1 | 1.51 | 0.9957 | 1 | 3 | 101.6(6.2) | 79.1(9.1) | 102.2(7.2) |
| Methiocarb | 1.36 | 1.34 | 1.63 | 0.9948 | 0.5 | 2 | 96.2(5.2) | 83.3(9.1) | 93.1(7.2) |
| Fenitrothion | 1.24 | 1.35 | 1.3 | 0.9967 | 1 | 3 | 102.1(7.3) | 80.2(8.7) | 109.3(7.2) |
| Pirimiphos-methyl | 1.09 | 1.13 | 1.2 | 0.9935 | 0.3 | 1 | 106.8(7.3) | 85.1(9.2) | 104.2(7.1) |
| Methiocarb sulfone | 1.05 | 1.55 | 1.43 | 0.9992 | 3 | 10 | 78.4(9.1) | 78.0(6.2) | 82.1(7.1) |
| Ethofumesate | 1.57 | 1.3 | 1.51 | 0.9993 | 3 | 10 | 73.4(7.1) | 74.2(9.3) | 79.1(9.5) |
| Linuron | 1.38 | 1.45 | 1.56 | 0.9999 | 1 | 3 | 73.2(9.2) | 78.3(8.2) | 79.7(4.3) |
| Probenazole | 1.03 | 1.49 | 1.64 | 0.9969 | 1 | 3 | 78.2(7.2) | 83.1(9.3) | 88.4(6.1) |
| Noruron | 1.22 | 1.77 | 1.79 | 0.9972 | 0.3 | 1 | 91.2(6.4) | 92.1(4.2) | 95.5(4.0) |
| Quinoclamine | 1.02 | 1.44 | 1.73 | 0.9926 | 0.3 | 1 | 103.8(7.1) | 104.1(4.0) | 97.2(6.3) |
| Dipropetryn | 1.27 | 1.16 | 1.42 | 0.9902 | 0.3 | 1 | 95.4(6.1) | 95.3(4.3) | 88.5(7.4) |
| Malathion | 1.19 | 1.4 | 1.18 | 0.9979 | 0.3 | 1 | 102.4(6.2) | 87.4(5.3) | 103.4(6.2) |
| Thiobencarb | 1.5 | 1.62 | 1.96 | 0.9903 | 0.5 | 2 | 94.3(9.2) | 84.3(5.4) | 94.3(6.2) |
| Diethofencarb | 1.4 | 1.19 | 1.77 | 0.9904 | 0.5 | 2 | 107.2(8.1) | 106.2(6.5) | 88.4(9.5) |
| Phorate sulfoxide | 1.21 | 1.13 | 1.39 | 0.9925 | 0.3 | 1 | 104.3(5.1) | 89.4(4.2) | 93.1(8.2) |
| Metolachlor | 1.26 | 1.47 | 1.64 | 0.9986 | 1 | 3 | 74.2(6.4) | 87.2(7.4) | 103.3(8.1) |
| Fenpropimorph | 1.21 | 1.36 | 1.27 | 0.9916 | 0.3 | 1 | 101.4(6.1) | 109.4(9.1) | 86.3(5.5) |
| Cyanazine | 1.11 | 1.24 | 1.7 | 0.9935 | 0.3 | 1 | 105.3(8.1) | 76.5(8.1) | 93.2(9.2) |
| Chlorpyrifos | 1.1 | 1.15 | 1.63 | 0.9989 | 0.3 | 1 | 94.5(7.2) | 110.2(8.1) | 97.2(5.2) |
| Parathion | 1.39 | 1.58 | 1.19 | 0.9942 | 1 | 3 | 107.1(7.5) | 87.3(7.4) | 105.1(7.1) |
| Flufenacet | 1.23 | 1.2 | 1.67 | 0.9978 | 1 | 3 | 101.3(5.2) | 79.2(9.1) | 102.1(5.2) |
| Rabenzazol | 1.13 | 1.67 | 1.4 | 0.9941 | 0.5 | 2 | 96.5(5.5) | 83.4(9.2) | 93.4(9.3) |
| 4,4'-Dichlorobenzophenone | 1.23 | 1.19 | 1.84 | 0.9902 | 1 | 3 | 104.3(7.4) | 80.5(9.6) | 109.2(7.3) |
| Triadimefon | 1.39 | 1.49 | 1.4 | 0.9927 | 0.3 | 1 | 107.4(7.4) | 85.5(9.3) | 104.1(7.2) |
| Chlorthal-dimethyl | 1.25 | 1.33 | 1.38 | 0.9945 | 1 | 3 | 95.1(6.3) | 93.2(9.1) | 94.5(6.4) |
| Dicapthon | 1.52 | 1.65 | 1.77 | 0.998 | 0.5 | 2 | 89.1(8.2) | 74.3(9.4) | 102.5(9.6) |
| Isofenphos-oxon | 1.15 | 1.48 | 1.46 | 0.9939 | 0.5 | 2 | 92.4(6.5) | 75.6(9.7) | 84.8(6.9) |
| Isocarbophos | 1.1 | 1.06 | 1.45 | 0.9924 | 0.5 | 2 | 85.0(7.1) | 107.2(9.3) | 123.4(9.5) |
| Tetraconazole | 1.03 | 1.12 | 1.46 | 0.9939 | 0.5 | 2 | 95.4(6.1) | 80.2(9.4) | 105.1(7.4) |
| Isobenzan | 1.19 | 1.5 | 1.28 | 0.9973 | 1 | 3 | 94.3(9.4) | 73.5(9.6) | 84.7(8.2) |
| Flurochloridone | 1.4 | 1.13 | 1.4 | 0.9943 | 1 | 3 | 84.6(7.3) | 75.8(9.2) | 94.0(8.1) |
| Fenson | 1.16 | 1.61 | 1.28 | 0.9976 | 0.3 | 1 | 90.5(8.4) | 99.5(9.1) | 105.2(4.3) |
| Pyracarbolid | 1.29 | 1.2 | 1.75 | 0.9949 | 0.5 | 2 | 105.3(7.1) | 81.2(9.5) | 106.5(7.4) |
| Dodemorph | 1.35 | 1.2 | 1.14 | 0.9925 | 1 | 3 | 92.5(6.6) | 90.4(4.2) | 94.5(5.3) |
| Mgk 264 | 1.42 | 1.42 | 1.73 | 0.9961 | 0.3 | 1 | 93.4(6.1) | 94.2(4.5) | 96.9(6.2) |
| Butralin | 1.55 | 1.35 | 1.74 | 0.9909 | 3 | 10 | 103.4(4.5) | 75.6(9.7) | 84.2(8.3) |
| Carbaryl | 1.13 | 1.47 | 1.21 | 0.994 | 0.3 | 1 | 104.2(7.3) | 87.4(8.2) | 101.4(5.3) |
| Diphenamid | 1.34 | 1.29 | 1.59 | 0.9958 | 0.5 | 2 | 109.5(7.6) | 92.7(4.8) | 93.3(8.2) |
| Pirimiphos-ethyl | 1.13 | 1.14 | 1.99 | 0.9997 | 1 | 3 | 97.4(9.5) | 84.6(9.7) | 108.8(9.3) |
| Isodrin | 1.4 | 1.51 | 1.49 | 0.9908 | 0.3 | 1 | 101.1(4.3) | 94.4(6.3) | 95.5(4.2) |
| Aldrin | 1.25 | 1.33 | 1.44 | 0.9915 | 1 | 3 | 85.3(4.4) | 84.5(8.1) | 107.3(5.2) |
| Isopropalin | 1.58 | 1.69 | 1.71 | 0.9983 | 0.5 | 2 | 94.2(8.2) | 97.1(4.4) | 108.6(8.3) |
| Cyprodinil | 1.06 | 1.09 | 1.98 | 0.9953 | 0.3 | 1 | 104.5(4.4) | 83.5(5.3) | 109.4(9.5) |
| Isofenphos-methyl | 1.55 | 1.41 | 1.52 | 0.9913 | 0.5 | 2 | 92.3(8.2) | 102.4(9.4) | 95.3(6.4) |
| Octachlorostyrene | 1.57 | 1.77 | 1.38 | 0.9922 | 0.3 | 1 | 107.5(9.4) | 96.5(7.1) | 88.3(4.2) |
| Metazachlor | 1.13 | 1.11 | 1.08 | 0.993 | 0.5 | 2 | 92.3(7.4) | 98.4(7.2) | 95.4(6.3) |
| Dimethametryn | 1.01 | 1.21 | 1.4 | 0.996 | 0.3 | 1 | 103.5(9.5) | 78.4(9.1) | 92.2(7.3) |
| Pendimethalin | 1.07 | 1.46 | 1.97 | 0.9924 | 3 | 10 | 110.3(8.1) | 97.2(9.4) | 96.3(7.3) |
| Disulfoton-sulfone | 1.45 | 1.64 | 1.61 | 0.9962 | 1 | 3 | 80.2(9.2) | 72.8(9.2) | 99.3(8.3) |
| Phorate sulfone | 1.16 | 1.34 | 1.32 | 0.9995 | 0.5 | 2 | 95.2(6.3) | 90.4(9.5) | 77.6(11.3) |
| Terbufos sulfone | 1.42 | 1.31 | 1.49 | 0.9939 | 1 | 3 | 96.5(5.4) | 80.3(4.3) | 109.5(9.6) |
| Paclobutrazol | 1.15 | 1.69 | 1.44 | 0.9958 | 3 | 10 | 95.5(8.6) | 92.3(8.5) | 95.5(9.0) |
| Penconazole | 1.01 | 1.8 | 1.08 | 0.9917 | 1 | 3 | 81.4(4.4) | 93.5(8.6) | 86.7(6.8) |
| Chlozolinate | 1.01 | 1.25 | 1.62 | 0.9969 | 3 | 10 | 95.1(6.3) | 97.2(7.4) | 96.5(8.6) |
| Pyrifenox | 1.17 | 1.14 | 1.46 | 0.9926 | 3 | 10 | 78.8(9.6) | 78.4(6.3) | 82.8(7.2) |
| Tolylfluanid | 1.24 | 1.44 | 1.25 | 0.9909 | 3 | 10 | 75.5(9.2) | 73.4(8.9) | 78.1(8.2) |
| Fosthiazate | 1.29 | 1.28 | 1.78 | 0.9906 | 1 | 3 | 82.8(5.1) | 89.3(2.2) | 89.2(5.3) |
| Phosfolan | 1.1 | 1.34 | 1.54 | 0.9985 | 1 | 3 | 88.2(8.4) | 82.3(9.3) | 93.2(6.1) |
| Allethrin | 1.45 | 1.3 | 1.51 | 0.9924 | 0.3 | 1 | 92.5(6.6) | 93.5(5.4) | 95.3(4.5) |
| Isofenphos | 1.53 | 1.64 | 1.97 | 0.9924 | 0.3 | 1 | 105.5(7.3) | 105.2(4.4) | 98.5(7.2) |
| Captan | 1.03 | 1.53 | 1.63 | 0.9999 | 0.3 | 1 | 95.3(6.1) | 95.6(4.4) | 88.5(7.2) |
| Fipronil | 1.05 | 1.47 | 1.76 | 0.9985 | 0.3 | 1 | 102.3(7.4) | 87.5(7.4) | 103.1(8.3) |
| Diclocymet | 1.22 | 1.17 | 1.51 | 0.9916 | 0.5 | 2 | 93.2(9.2) | 84.1(6.2) | 93.2(7.3) |
| Quinalphos | 1.23 | 1.58 | 1.63 | 0.9908 | 0.5 | 2 | 106.1(8.3) | 106.1(6.4) | 88.1(9.2) |
| Phenthoate | 1.49 | 1.58 | 1.61 | 0.9927 | 0.3 | 1 | 104.5(7.1) | 89.4(5.2) | 93.5(8.3) |
| Triadimenol | 1.5 | 1.17 | 1.81 | 0.9951 | 1 | 3 | 78.2(6.3) | 87.5(7.2) | 103.5(8.4) |
| Dinobuton | 1.45 | 1.62 | 1.37 | 0.9974 | 0.3 | 1 | 101.3(7.2) | 108.3(9.2) | 86.9(5.3) |
| Furalaxyl | 1.41 | 1.65 | 1.58 | 0.9971 | 0.3 | 1 | 105.3(8.2) | 76.8(9.1) | 93.4(8.2) |
| Crotoxyphos | 1.16 | 1.76 | 1.98 | 0.9992 | 0.3 | 1 | 94.5(7.1) | 112.6(8.2) | 97.7(5.3) |
| Procymidone | 1.11 | 1.05 | 1.08 | 0.9975 | 1 | 3 | 108.5(7.1) | 87.4(7.3) | 105.3(8.1) |
| Chlorbenside | 1.07 | 1.7 | 1.74 | 0.9966 | 1 | 3 | 101.2(6.2) | 79.1(9.1) | 102.2(7.2) |
| Chlorflurenol-methyl | 1.47 | 1.43 | 1.52 | 0.9934 | 0.5 | 2 | 96.2(5.2) | 83.3(9.1) | 93.1(7.2) |
| Chlordane | 1.09 | 1.22 | 1.19 | 0.9969 | 1 | 3 | 104.1(7.3) | 80.2(8.7) | 109.3(7.2) |
| Methidathion | 1.45 | 1.42 | 1.54 | 0.9919 | 3 | 10 | 75.4(9.1) | 78.0(6.2) | 82.1(7.1) |
| Haloxyfop-methyl | 1.07 | 1.5 | 1.36 | 0.9918 | 3 | 10 | 73.8(6.1) | 74.2(9.3) | 79.1(9.5) |
| Bromophos-ethyl | 1.07 | 1.71 | 1.83 | 0.99 | 1 | 3 | 74.2(8.2) | 78.3(8.2) | 79.7(4.3) |
| Procyazine | 1.14 | 1.14 | 1.15 | 0.9904 | 1 | 3 | 78.2(7.2) | 83.1(9.3) | 88.4(6.1) |
| Disulfoton-sulfoxide | 1.18 | 1.31 | 1.41 | 0.9908 | 0.3 | 1 | 94.2(6.9) | 92.1(4.2) | 95.5(4.0) |
| Tetrachlorvinphos | 1.48 | 1.58 | 1.4 | 0.9974 | 0.3 | 1 | 103.5(6.1) | 104.1(4.0) | 97.2(6.3) |
| Endosulfan | 1.2 | 1.29 | 1.36 | 0.996 | 0.3 | 1 | 95.4(6.1) | 95.3(4.3) | 88.5(7.4) |
| Mepanipyrim | 1.16 | 1.76 | 1.26 | 0.999 | 0.3 | 1 | 102.3(3.2) | 87.4(5.3) | 103.4(6.2) |
| Butachlor | 1.21 | 1.61 | 1.29 | 0.995 | 0.5 | 2 | 94.8(9.2) | 84.3(5.4) | 94.3(6.2) |
| Ditalimfos | 1.46 | 1.41 | 1.55 | 0.9952 | 0.5 | 2 | 105.2(8.1) | 106.2(6.5) | 88.4(9.5) |
| TCMTB | 1.12 | 1.15 | 1.36 | 0.9986 | 0.3 | 1 | 104.3(5.1) | 89.4(4.2) | 93.1(8.2) |
| trans-Nonachlor | 1.32 | 1.48 | 1.19 | 0.9912 | 1 | 3 | 74.2(6.4) | 87.2(7.4) | 103.3(8.1) |
| Chlorfenson | 1.4 | 1.67 | 1.57 | 0.9992 | 0.3 | 1 | 101.4(6.1) | 109.4(9.1) | 86.3(5.5) |
| Fenamiphos | 1.22 | 1.6 | 1.2 | 0.9912 | 0.3 | 1 | 105.3(5.1) | 76.5(8.1) | 93.2(9.2) |
| Picoxystrobin | 1.2 | 1.68 | 1.47 | 0.9919 | 0.3 | 1 | 94.5(7.2) | 110.2(8.1) | 97.2(5.2) |
| Napropamide | 1.19 | 1.5 | 1.26 | 0.9972 | 1 | 3 | 108.1(7.5) | 87.3(7.4) | 105.1(7.1) |
| Hexaconazole | 1.14 | 1.8 | 1.82 | 0.9943 | 1 | 3 | 103.3(5.2) | 79.2(9.1) | 102.1(5.2) |
| Flutolanil | 1.05 | 1.51 | 1.63 | 0.9906 | 0.5 | 2 | 96.5(5.5) | 83.4(9.2) | 93.4(9.3) |
| Prothiophos | 1.44 | 1.28 | 1.41 | 0.9928 | 0.3 | 1 | 105.3(7.4) | 80.5(9.6) | 109.2(7.3) |
| Isoprothiolane | 1.44 | 1.19 | 1.46 | 0.9914 | 0.3 | 1 | 106.4(7.4) | 85.5(9.3) | 104.1(7.2) |
| Profenofos | 1.54 | 1.18 | 1.69 | 0.9914 | 1 | 3 | 95.1(4.3) | 93.2(9.1) | 94.5(6.4) |
| tricyclazole | 1.43 | 1.73 | 1.35 | 0.9906 | 0.5 | 2 | 89.1(8.2) | 74.3(9.4) | 102.5(9.6) |
| Pretilachlor | 1.13 | 1.35 | 1.25 | 0.9986 | 0.5 | 2 | 92.4(6.5) | 75.6(9.7) | 84.8(6.9) |
| Dieldrin | 1.04 | 1.79 | 1.61 | 0.9965 | 0.5 | 2 | 85.0(7.1) | 107.2(9.3) | 123.4(9.5) |
| Oxadiazon | 1.59 | 1.59 | 1.72 | 0.9982 | 0.5 | 2 | 95.4(6.1) | 80.2(9.4) | 105.1(7.4) |
| Iprovalicarb | 1.03 | 1.11 | 1.08 | 0.9984 | 1 | 3 | 94.3(7.4) | 73.5(9.6) | 84.7(8.2) |
| Carboxin | 1.46 | 1.36 | 1.5 | 0.9922 | 1 | 3 | 84.6(8.3) | 75.8(9.2) | 94.0(8.1) |
| Myclobutanil | 1.53 | 1.56 | 1.55 | 0.9934 | 0.3 | 1 | 92.5(8.4) | 99.5(9.1) | 105.2(4.3) |
| p,p'-Dichlorodiphenyldichloroethylene | 1.16 | 1.22 | 1.68 | 0.9951 | 0.5 | 2 | 105.3(7.1) | 81.2(9.5) | 106.5(7.4) |
| Buprofezin | 1.13 | 1.14 | 1.39 | 0.9923 | 1 | 3 | 92.5(6.6) | 90.4(4.2) | 94.5(5.3) |
| Imazalil | 1.5 | 1.78 | 1.69 | 0.9965 | 0.3 | 1 | 93.4(6.1) | 94.2(4.5) | 96.9(6.2) |
| Flusilazole | 1.34 | 1.49 | 1.2 | 0.9995 | 3 | 10 | 103.4(4.5) | 75.6(9.7) | 84.2(8.3) |
| Methoprotryne | 1.3 | 1.34 | 1.12 | 0.9962 | 0.3 | 1 | 104.2(7.3) | 87.4(8.2) | 101.4(5.3) |
| Azaconazole | 1.43 | 1.36 | 1.61 | 0.9978 | 0.5 | 2 | 109.5(7.6) | 92.7(4.8) | 93.3(8.2) |
| Bupirimate | 1.4 | 1.15 | 1.92 | 0.9969 | 1 | 3 | 97.5(9.5) | 84.6(9.7) | 108.8(9.3) |
| Imazamethabenz-methyl | 1.54 | 1.66 | 1.89 | 0.9939 | 0.3 | 1 | 101.1(4.3) | 94.4(6.3) | 95.5(4.2) |
| Kresoxim-methyl | 1.46 | 1.5 | 1.66 | 0.9956 | 1 | 3 | 87.3(4.4) | 84.5(8.1) | 107.3(5.2) |
| Metamitron | 1.03 | 1.08 | 1.16 | 0.9999 | 0.5 | 2 | 94.2(8.2) | 97.1(4.4) | 108.6(8.3) |
| Isoxathion | 1.57 | 1.53 | 1.69 | 0.9921 | 0.3 | 1 | 106.5(4.4) | 83.5(5.3) | 109.4(9.5) |
| Aramite | 1.21 | 1.09 | 1.43 | 0.9942 | 0.5 | 2 | 92.3(5.2) | 102.4(9.4) | 95.3(6.4) |
| Nitrofen | 1.04 | 1.67 | 1.35 | 0.9951 | 0.3 | 1 | 107.5(8.4) | 96.5(7.1) | 88.3(4.2) |
| Endrin | 1.37 | 1.36 | 1.91 | 0.9926 | 0.5 | 2 | 96.3(7.4) | 98.4(7.2) | 95.4(6.3) |
| Endrin aldehyde | 1.32 | 1.78 | 1.26 | 0.9923 | 0.3 | 1 | 102.5(9.5) | 78.4(9.1) | 92.2(7.3) |
| Ancymidol | 1.05 | 1.27 | 1.7 | 0.9919 | 0.5 | 2 | 110.3(8.1) | 97.2(9.4) | 96.3(7.3) |
| Perthan | 1.42 | 1.47 | 1.36 | 0.9916 | 1 | 3 | 86.2(9.2) | 72.8(9.2) | 99.3(8.3) |
| Chlorfenapyr | 1.02 | 1.74 | 1.53 | 0.9963 | 0.5 | 2 | 95.1(6.3) | 90.4(9.5) | 77.6(11.3) |
| Chloropropylate | 1.22 | 1.52 | 1.12 | 0.9988 | 1 | 3 | 96.5(5.4) | 80.3(4.3) | 109.5(9.6) |
| Chlorobenzilate | 1.41 | 1.58 | 1.74 | 0.9948 | 3 | 10 | 95.5(7.6) | 92.3(8.5) | 95.5(9.0) |
| Fenthion sulfoxide | 1.39 | 1.11 | 1.42 | 0.9977 | 1 | 3 | 81.4(8.4) | 93.5(8.6) | 86.7(6.8) |
| Diniconazole | 1.53 | 1.67 | 1.15 | 0.9952 | 3 | 10 | 95.1(6.3) | 97.2(7.4) | 96.5(8.6) |
| Flamprop-isopropyl | 1.39 | 1.27 | 1.84 | 0.995 | 1 | 3 | 78.6(9.6) | 78.4(6.3) | 82.8(7.2) |
| p,p'-Dichlorodiphenyldichloroethane | 1.54 | 1.26 | 1.89 | 0.9919 | 3 | 10 | 75.5(9.2) | 73.4(8.9) | 78.1(8.2) |
| Aclonifen | 1.03 | 1.33 | 1.22 | 0.9916 | 1 | 3 | 82.7(5.1) | 89.3(6.2) | 89.2(5.3) |
| o,p'-Dichlorodiphenyltrichloroethane | 1.34 | 1.42 | 1.78 | 0.9965 | 1 | 3 | 88.2(8.4) | 82.3(9.3) | 93.2(6.1) |
| Oxadixyl | 1.26 | 1.3 | 1.85 | 0.9914 | 0.3 | 1 | 92.5(5.6) | 93.5(5.4) | 95.3(4.5) |
| Ethion | 1.4 | 1.73 | 1.97 | 0.9912 | 0.3 | 1 | 105.5(7.3) | 105.2(4.4) | 98.5(7.2) |
| Mepronil | 1.39 | 1.62 | 1.85 | 0.9957 | 0.3 | 1 | 95.3(6.1) | 95.6(4.4) | 88.5(7.2) |
| Triazophos | 1.17 | 1.48 | 1.53 | 0.9961 | 0.3 | 1 | 102.3(8.4) | 87.5(7.4) | 103.1(8.3) |
| Azamethiphos | 1.12 | 1.7 | 1.31 | 0.9963 | 0.5 | 2 | 93.9(9.2) | 84.1(6.2) | 93.2(7.3) |
| Ofurace | 1.36 | 1.41 | 1.46 | 0.9913 | 0.5 | 2 | 106.7(8.3) | 106.1(6.4) | 88.1(9.2) |
| Carbophenothion | 1.21 | 1.29 | 1.08 | 0.9988 | 0.3 | 1 | 104.5(7.1) | 89.4(5.2) | 93.5(8.3) |
| Benalaxyl | 1.49 | 1.49 | 1.54 | 0.9929 | 1 | 3 | 78.2(6.3) | 87.5(7.2) | 103.5(8.4) |
| Tepraloxydim | 1.38 | 1.43 | 1.92 | 0.9914 | 0.3 | 1 | 101.3(6.2) | 108.3(9.2) | 86.9(5.3) |
| Diofenolan | 1.04 | 1.31 | 1.55 | 0.9978 | 0.3 | 1 | 105.3(8.2) | 76.8(9.1) | 93.4(8.2) |
| Cyanofenphos | 1.13 | 1.75 | 1.67 | 0.9987 | 0.3 | 1 | 94.5(9.1) | 112.6(8.2) | 97.7(5.3) |
| Edifenphos | 1.3 | 1.52 | 1.65 | 0.9909 | 1 | 3 | 108.5(7.1) | 87.4(7.3) | 105.3(8.1) |
| Quinoxyfen | 1.02 | 1.12 | 1.08 | 0.9954 | 1 | 3 | 101.6(6.2) | 79.1(9.1) | 102.2(7.2) |
| Endosulfan Sulfate | 1.29 | 1.11 | 1.99 | 0.9999 | 0.5 | 2 | 96.2(5.2) | 83.3(9.1) | 93.1(7.2) |
| Propiconazol | 1.39 | 1.5 | 1.62 | 0.9926 | 1 | 3 | 102.1(7.3) | 80.2(8.7) | 109.3(7.2) |
| Norflurazon | 1.27 | 1.05 | 1.88 | 0.9997 | 3 | 10 | 78.4(9.1) | 78.0(6.2) | 82.1(7.1) |
| Fenhexamid | 1.35 | 1.36 | 1.26 | 0.9934 | 3 | 10 | 73.4(7.1) | 74.2(9.3) | 79.1(9.5) |
| p,p'-Dichlorodiphenyltrichloroethane | 1.15 | 1.79 | 1.28 | 0.9905 | 1 | 3 | 73.2(9.2) | 78.3(8.2) | 79.7(4.3) |
| Trifloxystrobin | 1.02 | 1.05 | 1.94 | 0.9931 | 1 | 3 | 78.2(7.2) | 83.1(9.3) | 88.4(6.1) |
| Hexazinone | 1.54 | 1.66 | 1.8 | 0.9978 | 0.3 | 1 | 91.2(6.4) | 92.1(4.2) | 95.5(4.0) |
| Tebuconazol | 1.18 | 1.34 | 1.49 | 0.9946 | 0.3 | 1 | 103.8(7.1) | 104.1(4.0) | 97.2(6.3) |
| Chloridazon | 1.05 | 1.8 | 1.75 | 0.9976 | 0.3 | 1 | 95.4(6.1) | 95.3(4.3) | 88.5(7.4) |
| Nuarimol | 1.28 | 1.78 | 1.36 | 0.9937 | 0.3 | 1 | 102.4(6.2) | 87.4(5.3) | 103.4(6.2) |
| Diclofop-methyl | 1.55 | 1.69 | 1.99 | 0.9945 | 0.5 | 2 | 94.3(9.2) | 84.3(5.4) | 94.3(6.2) |
| Triphenyl phosphate | 1.45 | 1.36 | 1.58 | 0.997 | 0.5 | 2 | 107.2(8.1) | 106.2(6.5) | 88.4(9.5) |
| Piperonyl butoxide | 1.09 | 1.49 | 1.53 | 0.9935 | 0.3 | 1 | 104.3(5.1) | 89.4(4.2) | 93.1(8.2) |
| Oxycarboxin | 1.03 | 1.44 | 1.88 | 0.9968 | 1 | 3 | 74.2(6.4) | 87.2(7.4) | 103.3(8.1) |
| Resmethrin | 1.5 | 1.41 | 1.59 | 0.9968 | 0.3 | 1 | 101.4(6.1) | 109.4(9.1) | 86.3(5.5) |
| Zoxamide | 1.13 | 1.65 | 1.35 | 0.9992 | 0.3 | 1 | 105.3(8.1) | 76.5(8.1) | 93.2(9.2) |
| Mefenpyr-diethyl | 1.11 | 1.74 | 1.84 | 0.9987 | 0.3 | 1 | 94.5(7.2) | 110.2(8.1) | 97.2(5.2) |
| Benzoylprop-ethyl | 1.39 | 1.41 | 1.91 | 0.9942 | 1 | 3 | 107.1(7.5) | 87.3(7.4) | 105.1(7.1) |
| Spiromesifen | 1.45 | 1.14 | 1.48 | 0.9922 | 1 | 3 | 101.3(5.2) | 79.2(9.1) | 102.1(5.2) |
| Endrin ketone | 1.14 | 1.65 | 1.64 | 0.9994 | 0.5 | 2 | 96.5(5.5) | 83.4(9.2) | 93.4(9.3) |
| Fenamiphos sulfone | 1.58 | 1.76 | 1.11 | 0.9945 | 1 | 3 | 104.3(7.4) | 80.5(9.6) | 109.2(7.3) |
| Bromuconazole | 1.56 | 1.18 | 1.88 | 0.9926 | 0.3 | 1 | 107.4(7.4) | 85.5(9.3) | 104.1(7.2) |
| Fenpiclonil | 1.06 | 1.14 | 1.2 | 0.9942 | 1 | 3 | 95.1(6.3) | 93.2(9.1) | 94.5(6.4) |
| Phosmet | 1.2 | 1.55 | 1.33 | 0.9998 | 0.5 | 2 | 89.1(8.2) | 74.3(9.4) | 102.5(9.6) |
| Bromopropylate | 1.04 | 1.24 | 1.84 | 0.9964 | 0.5 | 2 | 92.4(6.5) | 75.6(9.7) | 84.8(6.9) |
| Tetramethrin | 1.46 | 1.63 | 1.37 | 0.9903 | 0.5 | 2 | 85.0(7.1) | 107.2(9.3) | 123.4(9.5) |
| Picolinafen | 1.02 | 1.2 | 1.26 | 0.9962 | 0.5 | 2 | 95.4(6.1) | 80.2(9.4) | 105.1(7.4) |
| Bifenthrin | 1.27 | 1.62 | 1.56 | 0.9929 | 1 | 3 | 94.3(9.4) | 73.5(9.6) | 84.7(8.2) |
| Piperophos | 1.43 | 2.38 | 2.25 | 0.9996 | 1 | 3 | 84.6(7.3) | 75.8(9.2) | 94.0(8.1) |
| 4,4'-Methoxychlor | 1.01 | 1.5 | 1.57 | 0.9914 | 0.3 | 1 | 90.5(8.4) | 99.5(9.1) | 105.2(4.3) |
| Bifenazate | 1.07 | 1.33 | 1.34 | 0.9983 | 0.5 | 2 | 105.3(7.1) | 81.2(9.5) | 106.5(7.4) |
| Fenpropathrin | 1.5 | 1.65 | 1.77 | 0.9902 | 1 | 3 | 92.5(6.6) | 90.4(4.2) | 94.5(5.3) |
| Etoxazole | 1.04 | 1.48 | 1.92 | 0.9967 | 0.3 | 1 | 93.4(6.1) | 94.2(4.5) | 96.9(6.2) |
| Tebufenpyrad | 1.09 | 1.05 | 1.66 | 0.9908 | 3 | 10 | 103.4(4.5) | 75.6(9.7) | 84.2(8.3) |
| Fenamidone | 1.26 | 1.39 | 1.45 | 0.9971 | 0.3 | 1 | 104.2(7.3) | 87.4(8.2) | 101.4(5.3) |
| Dicofol | 1.28 | 1.58 | 1.31 | 0.9944 | 0.5 | 2 | 109.5(7.6) | 92.7(4.8) | 93.3(8.2) |
| Metconazole | 1.27 | 1.33 | 1.37 | 0.9952 | 1 | 3 | 97.4(9.5) | 84.6(9.7) | 108.8(9.3) |
| Fenazaquin | 1.09 | 1.34 | 1.67 | 0.9996 | 0.3 | 1 | 101.1(4.3) | 94.4(6.3) | 95.5(4.2) |
| Tetradifon | 1.58 | 1.8 | 1.93 | 0.9954 | 1 | 3 | 85.3(4.4) | 84.5(8.1) | 107.3(5.2) |
| Furathiocarb | 1.1 | 1.62 | 1.58 | 0.9919 | 0.5 | 2 | 94.2(8.2) | 97.1(4.4) | 108.6(8.3) |
| Phosalone | 1.17 | 1.28 | 1.26 | 0.9925 | 0.3 | 1 | 104.5(4.4) | 83.5(5.3) | 109.4(9.5) |
| Pyriproxyfen | 1.43 | 1.39 | 1.51 | 0.9983 | 0.5 | 2 | 92.3(8.2) | 102.4(9.4) | 95.3(6.4) |
| Mirex | 1.5 | 1.57 | 1.67 | 0.9904 | 0.3 | 1 | 107.5(9.4) | 96.5(7.1) | 88.3(4.2) |
| Mefenacet | 1.01 | 1.58 | 1.95 | 0.9916 | 0.5 | 2 | 92.3(7.4) | 98.4(7.2) | 95.4(6.3) |
| Cyhalothrin | 1.03 | 1.55 | 1.26 | 0.9934 | 0.3 | 1 | 103.5(9.5) | 78.4(9.1) | 92.2(7.3) |
| Tralkoxydim | 1.56 | 1.75 | 1.84 | 0.996 | 3 | 10 | 110.3(8.1) | 97.2(9.4) | 96.3(7.3) |
| Fenarimol | 1.34 | 1.72 | 1.55 | 0.9904 | 1 | 3 | 80.2(9.2) | 72.8(9.2) | 99.3(8.3) |
| Trifenmorph | 1.16 | 1.62 | 1.66 | 0.9981 | 0.5 | 2 | 95.2(6.3) | 90.4(9.5) | 77.6(11.3) |
| Azinphos-ethyl | 1.55 | 1.31 | 1.15 | 0.9967 | 1 | 3 | 96.5(5.4) | 80.3(4.3) | 109.5(9.6) |
| Pyrazophos | 1.58 | 1.34 | 1.46 | 0.9992 | 3 | 10 | 95.5(8.6) | 92.3(8.5) | 95.5(9.0) |
| Acrinathrin | 1.16 | 1.7 | 2.89 | 0.9996 | 1 | 3 | 81.4(4.4) | 93.5(8.6) | 86.7(6.8) |
| Fluoroglycofen-ethyl | 1.37 | 1.27 | 1.98 | 0.9977 | 3 | 10 | 95.1(6.3) | 97.2(7.4) | 96.5(8.6) |
| Fenoxaprop-ethyl | 1.12 | 1.52 | 1.69 | 0.9909 | 3 | 10 | 78.8(9.6) | 78.4(6.3) | 82.8(7.2) |
| Bitertanol | 1.15 | 1.23 | 1.48 | 0.9993 | 3 | 10 | 75.5(9.2) | 73.4(8.9) | 78.1(8.2) |
| Spirodiclofen | 1.33 | 1.58 | 1.45 | 0.9923 | 1 | 3 | 82.8(5.1) | 89.3(2.2) | 89.2(5.3) |
| Permethrin | 1.31 | 1.77 | 1.77 | 0.9995 | 1 | 3 | 88.2(8.4) | 82.3(9.3) | 93.2(6.1) |
| Pyridaben | 1.08 | 1.27 | 1.33 | 0.9987 | 0.3 | 1 | 92.5(6.6) | 93.5(5.4) | 95.3(4.5) |
| Fluquinconazole | 1.12 | 1.36 | 1.33 | 0.9972 | 0.3 | 1 | 105.5(7.3) | 105.2(4.4) | 98.5(7.2) |
| Coumaphos | 1.45 | 1.56 | 1.48 | 0.9987 | 0.3 | 1 | 95.3(6.1) | 95.6(4.4) | 88.5(7.2) |
| Prochloraz | 1.31 | 1.53 | 1.52 | 0.9997 | 0.3 | 1 | 102.3(7.4) | 87.5(7.4) | 103.1(8.3) |
| Butafenacil | 1.42 | 1.06 | 1.44 | 0.9977 | 0.5 | 2 | 93.2(9.2) | 84.1(6.2) | 93.2(7.3) |
| Prallethrin | 1.33 | 1.14 | 1.9 | 0.9979 | 0.5 | 2 | 106.1(8.3) | 106.1(6.4) | 88.1(9.2) |
| Cyfluthrin | 1.26 | 1.25 | 1.79 | 0.9946 | 0.3 | 1 | 104.5(7.1) | 89.4(5.2) | 93.5(8.3) |
| Cypermethrin | 1.37 | 1.43 | 1.42 | 0.997 | 1 | 3 | 78.2(6.3) | 87.5(7.2) | 103.5(8.4) |
| Boscalid | 1.01 | 1.53 | 1.61 | 0.9921 | 0.3 | 1 | 101.3(7.2) | 108.3(9.2) | 86.9(5.3) |
| Quizalofop-ethyl | 1.32 | 1.65 | 1.71 | 0.9933 | 0.3 | 1 | 105.3(8.2) | 76.8(9.1) | 93.4(8.2) |
| Flucythrinate | 1.11 | 1.52 | 1.55 | 0.9974 | 0.3 | 1 | 94.5(7.1) | 112.6(8.2) | 97.7(5.3) |
| Etofenprox | 1.35 | 1.38 | 1.82 | 0.9933 | 1 | 3 | 108.5(7.1) | 87.4(7.3) | 105.3(8.1) |
| Pyridalyl | 1.5 | 1.5 | 1.55 | 0.9936 | 1 | 3 | 101.2(6.2) | 79.1(9.1) | 102.2(7.2) |
| Fenvalerate | 1.03 | 1.68 | 1.84 | 0.9981 | 0.5 | 2 | 96.2(5.2) | 83.3(9.1) | 93.1(7.2) |
| Flumioxazin | 1.43 | 1.45 | 1.53 | 0.9956 | 1 | 3 | 104.1(7.3) | 80.2(8.7) | 109.3(7.2) |
| Pyraclostrobin | 1.05 | 1.47 | 1.73 | 0.9935 | 3 | 10 | 75.4(9.1) | 78.0(6.2) | 82.1(7.1) |
| tau-Fluvalinate | 1.03 | 1.41 | 1.97 | 0.991 | 3 | 10 | 73.8(6.1) | 74.2(9.3) | 79.1(9.5) |
| Difenoconazole | 1.41 | 1.65 | 1.61 | 0.9929 | 1 | 3 | 74.2(8.2) | 78.3(8.2) | 79.7(4.3) |
| Deltamethrin | 1.41 | 1.38 | 1.56 | 0.9916 | 1 | 3 | 78.2(7.2) | 83.1(9.3) | 88.4(6.1) |
| Azoxystrobin | 1.43 | 1.64 | 1.75 | 0.9974 | 0.3 | 1 | 94.2(6.9) | 92.1(4.2) | 95.5(4.0) |
| Dimethomorph | 1.52 | 1.61 | 1.95 | 0.9923 | 0.3 | 1 | 103.5(6.1) | 104.1(4.0) | 97.2(6.3) |

ME^a^: the ratio of the calibration curve slopes of Sin-QuChERS Nano column purification matrix to solvent.

ME^b^: the ratio of the calibration curve slopes of QuEChERS purification (complex matrix) to solvent.

ME^c^: the ratio of the calibration curve slopes of QuEChERS purification (simple matrix) to solvent.
